# Foraging complexity and the evolution of childhood

**DOI:** 10.1126/sciadv.abn9889

**Published:** 2022-10-12

**Authors:** Ilaria Pretelli, Erik Ringen, Sheina Lew-Levy

**Affiliations:** ^1^ Department of Human Behavior, Ecology and Culture, Max Planck Institute for Evolutionary Anthropology, Leipzig, Germany.; ^2^ Department of Anthropology, Emory University, Atlanta, GA, USA.; ^3^ Department of Comparative Cultural Psychology, Max Planck Institute for Evolutionary Anthropology, Leipzig, Germany.

## Abstract

Our species’ long childhood is hypothesized to have evolved as a period for learning complex foraging skills. Researchers studying the development of foraging proficiency have focused on assessing this hypothesis, yet studies present inconsistent conclusions regarding the connection between foraging skill development and niche complexity. Here, we leverage published records of child and adolescent foragers from 28 societies to (i) quantify how skill-intensive different resources are and (ii) assess whether children’s proficiency increases more slowly for more skill-intensive resources. We find that foraging returns increase slowly for more skill-intensive, difficult-to-extract resources (tubers and game), consistent with peak productivity attained in adulthood. Foraging returns for easier-to-extract resources (fruit and fish/shellfish) increase rapidly during childhood, with adult levels of productivity reached by adolescence. Our findings support the view that long childhoods evolved as an extended period for learning to extract complex resources characteristic of the human foraging niche.

## Teaser

Different foraging proficiency schedules for hard and easy foods suggest childhood evolved within a complex foraging niche.

## INTRODUCTION

Human childhoods are characterized by slow physical growth, extended dependence on parents and alloparents for provisioning, and increased investment in brain growth compared to nonhuman primates (*1*, *2*). Multiple hypotheses derived from life history theory have aimed to explain how this constellation of features was selected to maximize lifetime fitness. Following the dimensionless number model by Charnov *et al*. (*3*), which finds regular patterns of covariation between total life span and age at first birth across species, some researchers have suggested that human childhood is a by-product of our long total life spans (*4*). Other hypotheses posit that human childhood evolved alongside our increased reliance on the complex foraging niche typical for our species (*5*, *6*). Focusing on cooperation, the pooled energy model (*6*) argues that nonreproductive juveniles’ contributions to the family economy may have favored long childhoods. In this model, growing individuals adaptively shift their foraging patterns to target resources appropriate for their somatic and cognitive development. This generates an age-graded division of labor, through which children may reap direct fitness benefits by exchanging the resources they collect with those more effectively collected by others, and inclusive fitness benefits by helping close kin. Focusing on learning, the embodied capital theory (ECT) notes that difficult-to-acquire, energy-packed resources compose a large proportion of human diets (*5*). Collecting these resources requires high levels of coordination, strength, knowledge, and/or other cognitive skills. ECT hypothesizes that these traits—collectively termed “embodied capital”—are acquired during a protracted development. Under the assumptions of ECT, the costs associated with low productivity in early life and high rates of parental provisioning are offset by high lifetime productivity.

Several lines of empirical research using data from contemporary subsistence societies have aimed to test one of ECT’s key predictions: early life productivity should be low, with children’s foraging proficiency increasing with age alongside gains in knowledge, skill, and experience (*7*–*13*). Support for this prediction has been mixed. When considering overall caloric production, Kaplan *et al*. (*5*) found that among Hiwi, Ache, and Hadza, individuals only produce more than they consume in early to mid-adulthood. However, other studies have found that young children’s foraging returns can exceed their daily caloric needs. One 6-year-old Hadza forager reportedly produced 7000 kilocalories a day when collecting figs (*10*). Savannah Pumé children aged 11 to 14 can return 7500 kilocalories a day in fruit (*14*). Malagasy Mikea average 656 net kilocalories an hour when harvesting ovy tubers (*15*). In conflict with the expectation that more foraging experience should lead to greater foraging proficiency during childhood, time spent in boarding school (and thus away from foraging activities) did not negatively affect Hadza collection rates in an experimental task (*9*).

These mixed findings may be resolved by considering another of ECT’s key predictions: The difficulty of acquisition explains the age profile of production (*5*), with more difficult-to-acquire resources requiring longer investment in skill development. However, few studies have explicitly tested this prediction. Moreover, these have overwhelmingly focused on hunting, showing that large game hunting returns peak in mid-adulthood, several years after peak strength (*13*). This suggests that accumulated knowledge and experience related to understanding the natural environment (e.g., tracking and animal behavior), physical skill (e.g., aim and strength), and tool manufacturing (e.g., bows and poison) are fundamental to successful hunting (*11*, *16*). Still, children can achieve high returns by specializing in hunting matched to their size, skill, and strength. For example, Australian Martu children hunt goanna lizards in rocky outcrops, where they can maximize their returns given their height, stride length, and walking speed (*8*). Beyond hunting, young Mikea foragers preferentially target young ovy, whose tubers are small but shallow, and exploit patches more thoroughly than adults, in accordance with their smaller size and lesser strength. While Bird and Bird (*7*) found no effect of cognitive complexity on the age-specific production curves for various marine resources among the Australian Meriam, other authors qualitatively report that the timing at which foraging skills develop increases with task complexity. Both Hadza and Savanna Pumé children are described as becoming efficient in easier tasks, such as fruit collecting, before they effectively harvest tubers or hunt, which are more complex (*14*, *17*, *18*). Because much of this research is qualitative, from single populations and single resources, it is currently hard to assess whether observed variation in children’s foraging returns reflects cross-cultural differences in skill development, local foraging ecology, or study methodology.

Comparative analyses can help characterize how and why the life history of foraging varies across cultures. Data presented by Kaplan *et al.* (*5*) suggest that while Ache, Hiwi, and Hadza overall production increases with age, these trajectories are not uniform. In their studies of child foragers, Hawkes, Blurton Jones, and colleagues (*17*, *19*–*23*) argued that factors such as water availability, risk of getting lost, risk of predation, and availability of resources explained why Hadza children begin foraging so early, whereas San children begin much later. In the largest comparative study of hunting to date, Koster *et al.* (*13*) found that in 40 societies overall skill peaked in adulthood, although there was considerable inter- and intrasocietal variation in age-specific returns. Because only 7% of observations in this study came from individuals younger than 20, and a mere 0.2% from children younger than 10, the ontogeny of hunting skill in early life remains poorly characterized. Furthermore, we do not yet know how hunting skill development compares to that of other resources exploited by humans.

Although the human foraging niche may be generally more complex than that of other primates, the foods that foragers pursue are not uniformly difficult to collect. Complexity can vary along two major axes of strength and knowledge (*14*), and each task within the human ecological niche requires a specific set of competencies along these axes, which are acquired at variable time lines (*24*,*25*). For example, to successfully collect fruit, a forager must be coordinated, know where to find ripe fruit, and, in some cases, have the agility and strength to climb tall trees (*10*). Collecting tubers requires yet more skill: Underground storage organs (USOs) are usually embedded deep in hard substrate. A forager must have the knowledge to locate the tuber, the strength to excavate it, and the skill to make and use appropriate tools (*9*). Food items embedded in hard substratum, mobile prey, or food products requiring specialized technologies for the most part require high levels of both strength and knowledge for successful extraction (*26*, *27*). To investigate whether complex resources are associated with slower learning curves and, thus, the evolution of longer childhoods, research is needed to quantify variation in the ontogeny of foraging productivity according to resource type complexity.

**Fig. 1.**
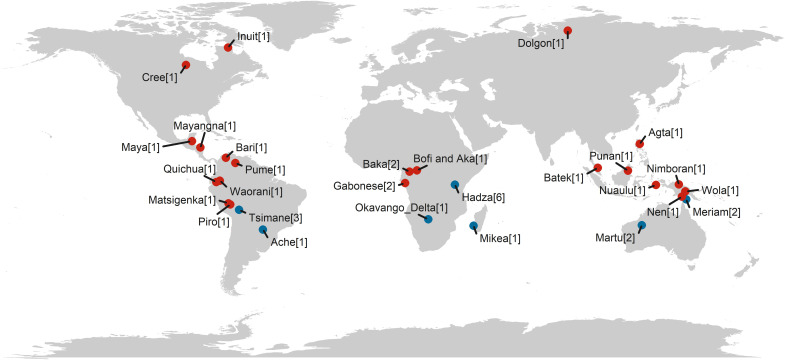
Populations. World map indicating the locations of study populations included in the present study. The numbers of datasets for each population are in square brackets. The most represented community, the Hadza, appears in six studies. Populations for which datasets were sourced from the literature are in blue. Populations for which datasets were sourced from Koster *et al.* (*13*) are in red.

**Fig. 2.**
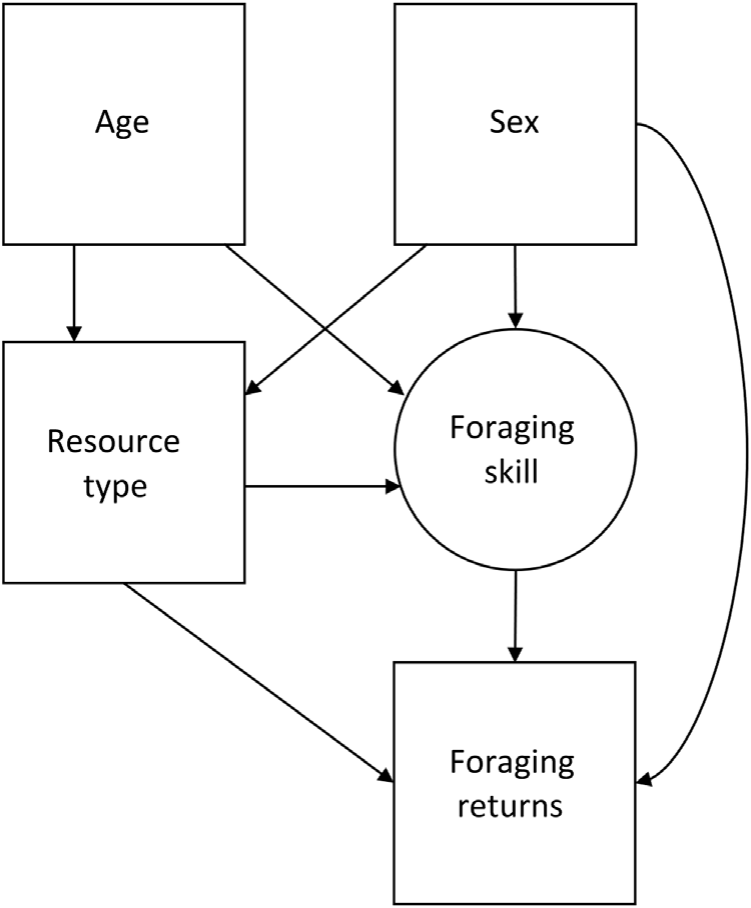
Directed acyclic graph. This DAG illustrates the proposed causal associations between the main factors investigated in this analysis. Age and sex affect the probability of choosing a resource. Skill levels also depend on age and sex. Returns vary across resources and depend on skill. Thus, all effects of age pass through skill (the sum of all age-varying traits that influence returns), excluding the influence that age has on the choice of resource (i.e., children perform different activities at varying ages).

In the present study, we aimed to test ECT’s prediction that the development of foraging proficiency is slower for more complex resources. We operationalize foraging proficiency as age-specific foraging returns. We compiled a dataset from published sources on foraging returns, totaling observations for 714 children and adolescents from 28 societies (see Fig. 1). We consider four resources differing in complexity: fruit and marine resources, which require less strength and individual knowledge to collect, and game and USOs (i.e., tubers), which require specialized tools, knowledge, and strength. Using these data, we model resource-specific foraging returns as a function of individual skill, a dimensionless latent variable that varies with age and sex (Fig. 2). Skill summarizes all traits relevant to foraging, thus conceptually referring to a combination of cognitive and physical embodied capital such as knowledge and strength. This allows us to (i) assess whether children’s proficiency increases more slowly for more complex resources and to (ii) quantify the skill intensity for resources varying in complexity, i.e., how much underlying skill is needed to successfully forage a certain resource. Our approach can thus help resolve outstanding ambiguity regarding children’s foraging proficiency and skill ontogeny.

## RESULTS

### Age-specific foraging returns

**Fig. 3.**
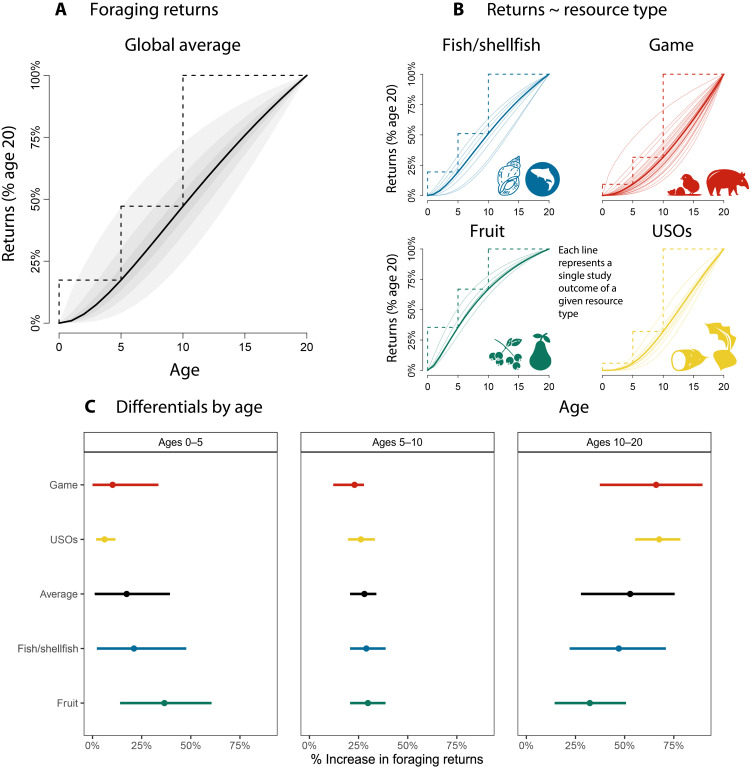
Foraging returns. (**A**) Predicted change in foraging returns with age, averaging over variation between studies, individuals, sex, and resource type. The *x* axis shows age, while the *y* axis is a unit-free measure of the proportion of increase compared to the maximum value (predicted returns at age 20). Solid line is the posterior median prediction; shaded intervals depict the 30th, 60th, and 90th percentile credible intervals. Dashed lines highlight arbitrary age differentials across childhood. (**B**) Predicted change in foraging returns by resource type, with thick lines denoting the average posterior median and thin lines denoting the median for each unique study outcome for that resource type. All curves are scaled by their maximum value (predicted returns at age 20). The shape of the curves illustrates how productivity increases with age. (**C**) Percentage increase in foraging returns across childhood, covering the intervals denoted by the dashed lines in (A) and (B). Points indicate posterior median, and bars indicate 90% HPDI (Highest Posterior Density Interval).

We found that, in general, foraging returns increase steadily throughout childhood and adolescence (Fig. 3A). By age 5, the average child has achieved about 20% of the productivity that they will have achieved by age 20. This value increases to approximately 50% by age 10. The largest increase in foraging returns happens between 10 and 20 years of age.

### Resource-specific development of foraging proficiency

The general pattern of proficiency increase varies across resource type (Fig. 3B): Game and USOs exhibit accelerating returns, and fruit shows diminishing returns with age. Fish/shellfish exhibit an intermediate pattern. The greatest gains happen early in life for fruit, while gains in game and tubers continue into adolescence and likely peak only in later adulthood (Fig. 3C).

### Skill intensity of resources

**Fig. 4.**
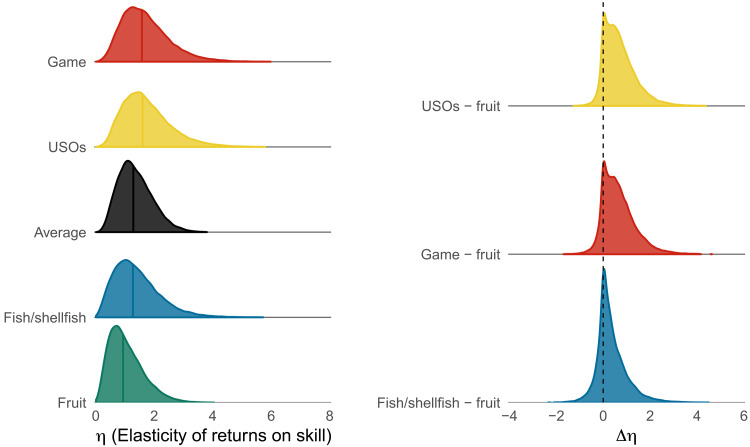
Skill intensity of resources. (**Left**) Posterior distributions for the elasticity of foraging returns on skill (η) for each resource type. Higher values indicate that returns are more dependent on skill. (**Right**) Contrast between the elasticity of USOs, game, and fish/shellfish versus fruit resources, showing how much more skill intensive these resources are (posterior probability η USOs *>* η fruit = 0.89, η game *>* η fruit = 0.86).

Figure 4 shows the posterior distribution for η, which is the parameter indicating skill intensity. η provides an indication of how foraging returns relate to skill, that is, whether increasing skill results in diminishing or accelerating foraging returns. η is the elasticity of skill and controls its effect on returns, represented by the arrow that connects skill to returns in Fig. 2. The four types of resources analyzed here differ in how skill intensive they are, with game and tubers requiring more skill, fruit requiring less skill, and fish/shellfish in between (Fig. 4, left). The maximum difference in skill intensity is between USOs and fruit: Figure 4 (right) shows that values of η relative to USOs are greater than those of fruit in about 90% of the posterior samples, indicating with substantial confidence that extracting tubers is more skill intensive than collecting fruit. Game shows a similar pattern, with 86% of posterior samples for η relative to game greater than η values for fruits. Note that there is considerable heterogeneity across studies within each category. In particular, “game” resources span a wide array of foraging skill and return curves, each implying distinct life history trajectories (Fig. 3B). Thus, we should be cautious in making strong claims about categorical differences between hunting and other types of foraging.

### Sex differences

There are minimal gender differences in skill development, with skill appearing to increase slightly faster in early ages for males than for females (fig. S2). Similarly, there were no strong resource-specific sex differences, apart from females showing more variation in the age-specific returns between resources, whereas males show similar patterns across resources (see figs. S3 and S4). However, these findings should not be interpreted as evidence for no sex differences, given how uncertain our prediction intervals are. Instead, it may be that our data are of insufficient resolution to detect differences between male and female foragers—which are most likely smaller than differences between resource types.

## DISCUSSION

Childhood has been theorized to have evolved as an extended learning period for collecting complex resources. However, no studies to date have explicitly modeled the association between resource complexity and children’s productivity in a cross-cultural sample. Here, we empirically estimate how skill intensive different resources are and investigate age-specific returns for these resources. By testing predictions from the ECT, our findings have important implications for current debates regarding the evolution of childhood and point to several avenues for future research.

We found that by 10 years of age, children in our sample achieved half of the productivity of 20-year-olds. Echoing research among Hadza, Mardu, Maya, BaYaka, Aka, and communities in the Okavango Delta (*8*, *10*, *28*–*31*), these findings suggest that children can be independent foragers well before maturity, given the right socioecological conditions. Still, in general, foraging returns continue to increase at least until adulthood for all resources, suggesting that individuals acquire embodied capital throughout the entire prereproductive stage (*5*).

The complexity of our species’ foraging niche is reflected in our findings. For most datasets included in the present analysis, η is estimated to be greater than 1 (see fig. S6). This means that gains in foraging proficiency are dependent on considerable gains in skill. Within taxa, niche complexity and life history traits, such as length of development, are correlated (*26*). The foraging niches of other primates include a larger proportion of resources that are expected to have lower skill intensity. For example, chimpanzees are known to hunt small prey and eat nuts or tubers, but they are overall ripe-fruit specialists (*32*). While baboons are considered generalists, they still rely on fruit and leaves for most of their diets (*33*). Thus, while these species do collect complex resources, they do not specialize in them. Humans, on the other hand, preferentially target complex resources (*5*, *34*), and this complexity is reflected across almost all sampled societies in the present analysis.

Our model estimated that tubers and game are more skill-intensive resources to collect, compared to fish/shellfish and fruit. Children’s productivity varied considerably by resource type: Returns for more skill-intensive resources peaked later than those for less skill-intensive resources. In other words, children reached adult levels of proficiency for fruit early in life, whereas proficiency for tubers and game continued to increase into adulthood. These differences were better explained by variation in skill intensity (Fig. 4) than by the timing of skill acquisition (fig. S5). “Skill,” a key parameter in our analysis, is an abstraction that captures differences in foraging ability with age and between individuals, but it has a nonlinear relationship to actual returns. Some resources, like fruit, exhibit diminishing returns to skill, implying that even the best fruit collector will not produce much more than average, possibly due to constraints such as carrying weight and distance between patches. In contrast, resources such as game exhibit accelerating returns, with relatively low expected returns at low and moderate levels of skill. In summary, our findings suggest that children’s foraging proficiency is dependent on skill acquisition, with foraging proficiency for more complex resources requiring longer periods of skill development. These findings support the view that complex resources require a longer investment in learning and thus, in line with ECT, may have promoted the evolution of childhood.

Our analysis allowed us to explicitly estimate skill. This measure likely reflects various individual traits that contribute to skill, including somatic and cognitive traits such as coordination, endurance, agility, wayfinding, traditional ecological knowledge, problem solving, and planning. An implication of ECT is that cognitive more than somatic traits are the limiting factor when foraging complex resources. In their research with Mardu and Meriam, Bird and Bird (*7*, *8*) find that foraging performances are largely constraint by size. However, knowledge was not explicitly measured in their analyses. Considering that size, strength, and knowledge tend to develop together (*24*), it remains unclear whether their findings are at odds with, or complementary to, predictions derived from ECT. Similarly, and because few studies consistently report individual measures for size, strength, and knowledge, our measure for skill does not differentiate between different types of embodied capital. Instead, our findings suggest that to target complex resources, children require high levels of skill, which they acquire through an unknown combination of learning and growing. Nonetheless, the difference observed across resource complexity is consistent with the coevolution of early life history traits and our especially complex foraging niche. We call on researchers to collect data on various aspects of embodied capital to tease apart their relative contribution to foraging skill across resource types.

This paper has several limitations related to the comparability of the data that we used. There were considerable differences in how data were collected, whether returns were presented as quantities or rates, and whether data were for individuals or for age classes. This could compromise some of our inferences as data collected with different methods could be biased toward or against nonzero returns. However, fig. S7 indicates that our model is able to predict the proportion of nonzero returns with sufficient accuracy. Furthermore, across datasets, trip level traits such as travel time and group size are differently accounted for. Unfortunately, we could not address this problem statistically, as the relevant details of the data collection procedures (i.e., treatment of zero return trips) were highly variable and often not reported. In addition, these study-specific parameters are highly correlated with resource type: With few exceptions, each study reports returns for only a single category of resource. This makes it difficult to confidently assess whether variation is due to true differences between resources or to unmeasured differences between populations or in study methodology. This issue is especially apparent for shellfish, where the large majority of the available data comes from one research group (*7*, *24*, *35*). Our combined dataset also contains few repeated measures, which made it impossible to model individual variation in the ontogeny of foraging skill. Our analysis also highlights limitations inherent to the available literature. Overreliance on cross-sectional data leaves us vulnerable to misinterpreting cohort effects for age effects. Hence, longitudinal datasets of foraging returns that span several decades are needed (*13*). Furthermore, knowledge, strength, size, and cognition all likely vary independently and contribute differently to resource-specific skill. For example, Bird and Bird (*8*) found that Mardu children’s walking speed is a good predictor of goanna lizard hunting success. For Tsimane hunters, the ecological knowledge needed to directly encounter animals had the biggest effect on individual hunting returns (*36*). Moreover, available data mostly focus on either children or adult foraging returns, making it difficult to develop continuous measures of skill development through the whole life span. To better understand the resource-specific development of skill beyond the general estimation presented here, future studies should integrate ethnographic understanding of each population’s subsistence strategies and individual-level measures of traits that may contribute to skill. Future studies should also consider heterogeneity in complexity within resource types across regions, seasons, and based on available extractive technologies. For example, while we considered hunting more generally, prey types vary by size, seasonal abundance, distribution, and the availability of efficient hunting technologies. Future studies should consider this variation when reporting on hunting returns.

Finally, although our study focused on ECT, our findings are also compatible with the pooled energy model. While much research into human social organization has focused on the gendered division of labor, the coordination of work between children and adults may be equally important (*37*–*39*). As Kramer (*40*) points out, adults and children participate in bidirectional exchanges characterized by labor specialization and food sharing (*6*). Our results suggest that children may specialize in fruit and fish/shellfish collection early on, even as they continue to gain skill in more complex tasks as they grow. Furthermore, social learning and social foraging can also scaffold children’s participation in food production, even if they have not yet acquired all underlying skills (*41*). For example, children can help identify tuber vines before they are strong enough to collect them themselves. With the help of more experienced foragers, children can harvest ripe fruits even when they do not know where to find them. To fully understand variation in age-specific foraging returns and to better assess predictions from the pooled energy model, future studies should move beyond measuring only individual returns and toward accounting for how children coordinate their labor with other household and community members.

To conclude, we found that children’s age-specific foraging proficiency varies with resource-specific skill intensity. In support of ECT, this finding is consistent with the view that long childhoods evolved as an extended period to learn to exploit the most complex resources in our foraging niches. Our analysis also suggests that unmeasured factors related to individuals and their social and ecological settings may also contribute to variation in foraging returns across resource types and cultures (*28*). Such factors, including individual motivation, social networks, social foraging, and resource availability, for the most part remain underreported in the existing literature despite their importance to understanding how long human childhoods generally, and children’s participation in foraging specifically, coevolved alongside our species’ propensity for cooperation and cumulative culture. To fully understand the developmental trajectories of children’s foraging returns and their articulation with ecological and social contexts in the present and throughout our evolutionary history, we call on researchers to consider these variables in their future research.

## MATERIALS AND METHODS

### Study selection

We followed a systematic two-step protocol for locating relevant published articles, as summarized in fig. S1. First, we queried major search engines (Google Scholar, JSTOR, PsycNet, ScienceDirect, Springer, and Wiley) with the keywords ‘children’&‘foraging’&‘returns’ on 26 September 2019. This search produced 360 unique papers. After reading abstracts for eligibility, 133 papers were read in full. Thirty-five papers were identified as potentially including data on foraging returns from children, according to two independent coders. Second, we endeavored to locate additional relevant texts. We searched through the bibliographies of papers with relevant data identified during our initial search. We also looked through the publication list of the first authors of these papers. We repeated these steps for all newly identified relevant papers. This search method yielded a total of 40 papers potentially containing data on children foraging returns.

We screened these studies against our inclusion criteria: (i) the paper reported original data on foraging returns from children and/or adolescents (time allocation studies, secondary analyses, and reviews were not eligible for inclusion); (ii) the paper contained individual-level data or group-level means and variances; (iii) the paper reported data for multiple prereproductive individuals or age groups (reports of returns for single prereproductive age groups were excluded); (iv) the data were presented as continuous quantities, e.g., kilocalories per day and grams per hour. Ranges were not eligible. For example, Kawabe (*42*) reports number of animals (1 to 5 or more than 5) by species killed across childhood. This study was not included in the present analysis. Finally, (v) we included data for individuals and age groups 20 years and under. If the age range of an age group crossed 20 years, this age group was excluded. This was due to the fact that research focusing on children foraging often does not report adult foraging returns, and considering age groups that span both adolescence and adulthood would complicate interpretation.

To identify studies that used the same data in separate analyses published in different papers, we compiled metadata for each paper. Datasets were considered overlapping if they were collected in the same population, time period, and for the same set of resources. Age ranges, reported data collectors, unit of measure (e.g., kilocalories), and methods of data collection (e.g., naturalistic or experimental) were also examined. See table S1 for more details on metadata for the selected papers. In cases where reports of children’s foraging returns were duplicated, we retained the paper with the most detailed information, such that individual returns were preferred over group returns, and reports with specific ages were preferred over those focused on age classes. We also included child hunting return data available in the *cchunts* package from Koster *et al.* (*13*). Two papers contained data present in the *cchunts* package and were hence discarded. A total of 38 papers that contain 58 datasets produced a sample of 714 individuals and group measures from 28 societies on five continents (one to six studies per society—mean, 1.39; SD, 1.03; see Fig. 1).

### Coding

Data presented in tables were extracted by transcribing the values. Data presented in figures were extracted using the metadigitalize R package (*43*). Two coders independently extracted each type of data. Values were compared and averaged to account for potential coder error. Data from the *cchunts* R package present individual and pooled hunting returns. Of these, we selected all the observations for individuals below 20 years of age, which represent 70% of our data. Supplementary analyses suggest that the inclusion of pooled hunting returns do not skew the results (see fig. S13). We assigned a targeted resource to each dataset based on information present in text and figure captions of the papers. Most papers contained data referring to a single resource, e.g., hunting returns for game or fish and shellfish. In cases where a paper contained different resources types, we unpacked the data, treating data points relative to different resources as different outcomes. If data points could not be attributed to specific resources, they were categorized as mixed, as were data relative to eggs and honey. These “mixed” data contributed to the estimation of posterior values for the overall estimates but not the specific resource comparisons. We followed Johnson and Bock (*44*), Lancaster *et al.* (*25*), Schuppli *et al.* (*26*), and Kramer (*14*) in categorizing resource complexity according to the degree to which strength and knowledge were required for successful extraction. In this framework, foods that are sessile and can be simply collected (e.g., fruit) require less strength and knowledge for collection, whereas those that need to be extracted from a hard substrate (e.g., USOs) and food that moves and needs to be hunted down (namely, game) require high levels of strength and knowledge for collection. We thus defined game and USOs (i.e., tubers) as more complex and fruit and fish/shellfish as less complex. Note that because it is rarely reported in the published literature, we were not able to account for variation in game size, although we acknowledge that there may be substantial differences in skill development for small and large game. Note as well that shellfish is here considered a collected resource as we did not account for the complex processing phase. All data points are represented in figs. S8 to S12.

### Statistical model

Following recent studies on the ontogeny of subsistence knowledge and ability (*13*, *30*), we used a dynamical model of foraging that allowed us to estimate how foraging skill accumulates with age and how skill (a latent variable) relates to observed returns (which vary for reasons other than forager skill). We used a hurdle model to describe both the probability of acquiring any return at all and probability of harvesting a certain amount of resources. Assume that individuals go on foraging trips in which they successfully acquire some return (*y* > 0) with probability *p* or come home empty-handed (*y* = 0) with probability 1 − *p*. Furthermore, assume that nonzero returns follow a log-normal distribution. Observed foraging returns are thus mapped onto a hurdle model where

f(y)=Bernoulli(1−p) if y=0 (1)

$$\begin{array}{cc} f(y)=p[\text{LogNormal}(\mu ,\sigma )] & \text{if}\;y>0 \end{array}$$ (2)

Previous studies of human foraging returns have found that both the probability of a zero return and the quantity of returns depend on forager skill (*S*), which varies across the life span. As a directed acyclic graph, this can be conceived of as age → *S* → *p* and *S* → μ (see Fig. 2). Koster *et al.* (*13*) modeled the relationship between age and *S* as a concave downward function to account for senescence among older adults. However, our focus was on the returns of foragers below age 20—more than a decade earlier than the estimated peak of foraging skill—so we did not model senescence. Otherwise, we used the same functional form as Koster *et al.* (*13*) and Lew-Levy *et al.* (*30*) to describe change in latent foraging skill with age

$$S(\text{age})={\left[1-\text{exp}(-k\times \text{age})\right]}^{b}$$ (3)

where *k* is the constant rate of growth in foraging skill and *b* is an elasticity parameter that determines the proportional change in skill. Skill itself has nonlinear effects on foraging success.

Depending on how “difficult” the subsistence task is, skill may be more or less important for actual foraging productivity, which we model with an additional elasticity parameter η. η < 1 indicates diminishing returns (decreasing differentials of returns with increasing skill), while η > 1 indicates accelerating returns (increasing differentials of returns as skill increases). Comparison of η thus offers empirical estimates of skill intensity for different types of resources (e.g., fruit versus game). *k*, *b*, and η were assumed to be positive, which means that skill is strictly increasing with age and that higher skill always has a positive effect on foraging returns. Finally, we add the log-linear α, which acts as an intercept for foraging returns, independent of age

$$\mu =\text{log}(S^{\eta \mu }\alpha_{\mu })$$ (4)

$$p=2[{\text{logit}}^{-1}(S^{\eta p}\alpha_{p})-\frac{1}{2}]$$ (5)

**Fig. 5.**
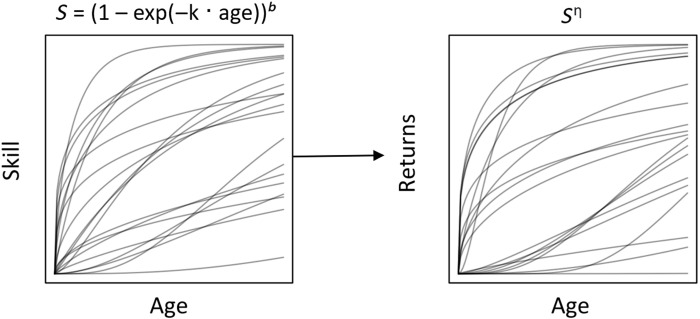
Combined priors. Curves depict possible curves for skills and foraging returns given the weakly regularizing priors in the model.

Figure 5 shows prior distributions of skill and corresponding returns. We used weakly regularizing priors, as described in the Supplementary Materials, so that multiple possible shapes of the correlation between both skill and returns with age are possible, allowing sufficient flexibility to comfortably fit any effect of age.

To untangle the effect of age from that of other factors, our model was multilevel, which allowed us to accommodate variation across individuals, sex, resource type, and study. We allowed the parameters *k*, *b*, η, and α to vary among and between studies (i.e., if a single study had multiple outcomes) and resource type using random effects

$$\text{log}(\alpha )=\alpha 0+v\alpha [{\text{outcome}}_{]}+v\alpha [\text{resource}]+v\alpha [\text{sex}]+v\alpha [\text{sex}:\text{outcome}]+v\alpha [\text{sex}:\text{resource}]$$ (6)

$$\text{log}(k)=k_{0}+v_{k[\text{outcome}]}+v_{k[\text{resource}]}+v_{k[\text{sex}]}+v_{k[\text{sex}:\text{outcome}]}+v_{k[\text{sex}:\text{resource}]}$$ (7)

$$\text{log}(b)=b_{0}+v_{b[\text{outcome}]}+v_{b[\text{resource}]}+v_{b[\text{sex}]}+v_{b[\text{sex}:\text{outcome}]}+v_{b[\text{sex}:\text{resource}]}$$ (8)

$$\text{log}(\eta )=\eta_{0}+v_{\eta [\text{outcome}]}+v_{\eta [\text{resource}]}+v_{\eta [\text{sex}]}+v_{\eta [\text{sex}:\text{outcome}]}+v_{\eta [\text{sex}:\text{resource}]}$$ (9)

We also model correlations between the random (varying) effects *v* to account for the possibility that studies where the base rate of skill acquisition is higher may have lower age-independent returns. To account for repeated measures of participants in some studies, we also included a random intercept for skill across individuals.

We accounted for measurement error in forager age, which can lead to deflation of parameter estimates, i.e., attenuation bias, by replacing the extracted ages, which were given as either point estimates or age intervals, with a Gaussian measurement error model. We highlight that all model parameters are estimated jointly, from a combination of eight chains with 6000 iteration steps each.

All analyses were run in R (version 4.2.0), and all models were fit using the RStan package (Stan Development Team 2020), which fits Bayesian models using Hamiltonian Markov chain Monte Carlo. Markov chain convergence was assessed using standard diagnostics (number of effective samples, the Gelman-Rubin diagnostic, and visual inspection of trace plots). More details on the model can be found in section S1.2.

We used posterior samples drawn from our model to predict foraging returns given different combinations of age, resource type, and sex. These predictions are dimensionless quantities of productivity that are only interpretable in relative terms (as opposed to, for example, a rate with dimensions like kilocalories per hour). As such, we cannot say whether children in one society are more skilled than another or whether girls are more productive than boys. We can only determine how the shape of the age trajectories vary. While modeled as a continuous measure, we quantified age-specific pattern by assessing foraging relative to the return quantity predicted for a 20-year-old (the oldest age included in our dataset). This takes the form $\frac{\text{model prediction at age}\;x}{\text{model prediction at age}\;20.}$

## Supplementary Materials

### This PDF file includes:

Supplementary Text Table S1 Figs. S1 to S15 References

## Notes

View/request a protocol for this paper from *Bio-protocol*.
