# Supplementary material for: Foraging complexity and the evolution of childhood
Source: Sci Adv. 2022 Oct 12;8(41):eabn9889. doi: 10.1126/sciadv.abn9889 (PMC9555775; doi:10.1126/sciadv.abn9889)
Supplement: Supplementary file 1 — Supplementary Text Table S1 Figs. S1 to S15 References [file sciadv.abn9889_sm.pdf]

Science Advances
AAAS

Supplementary Materials for

Foraging complexity and the evolution of childhood

Ilaria Pretelli *et al.*

Corresponding author: Ilaria Pretelli, ilaria_pretelli@eva.mpg.de

Sci. Adv. **8**, eabn9889 (2022)
DOI: 10.1126/sciadv.abn9889

This PDF file includes:

Supplementary Text
Table S1
Figs. S1 to S15
References

1 Supplementary Information

1.1 Data sets and metadata

In figure S1 we summarize the literature selection process that produced our sample. Table S1 shows some metadata referring to the datasets included in the analysis. The data are a combination of datasets obtained directly from the literature, the first 11 rows in the table below, and original data organized by Koster et al. (*13*) in the `cchunts` package, marked by the code `cchunts` in the Data column. Specifically, Koster "searched for relevant studies on subsistence hunting in the anthropological and biological literature, subsequently contacting authors to invite them to contribute data. The contributors submitted data in a standardized format that included variables for the biomass acquired on terrestrial hunting trips, the ages of the hunters at the time of the hunt, the duration of the trip, the hunting weaponry carried by the hunters, and the presence of dogs or assistants" (*13*).

Following the inclusion of data from this source, we screened the data we extracted from published papers to remove repeated data sets. In particular, data relative to the Ache of Paraguay extracted from Walker et al. (*16*) have not been used because they are already present in the "Hill_Kintigh" data set included in the `cchunts` package. Moreover, Tsimane data extracted from Gurven et al. (*11*) are a repetition of those included in the `cchunts` data ("Trumble_Gurven"). Only the latter were used in the analysis.

Data collected by Bliege Bird and Bird among the Martu in Western Australia come from both a 2005 study on children's foraging (*8*) and from the dataset in the `cchunts` package (*13*). The 2005 paper reports data from individual of both sexes between 5 and 14 years old hunting *goanna* lizards in the rocky outcrop not far from the camp. These data were collected by the authors between 2000 and 2002. The `cchunts` data were collected between 2002 and 2010, are relative to individuals aged 7 to 79 and partially exclude female contributions ("This data set includes observations of female foragers when they were accompanied by men on trips, but not women on foraging trips that did not include male foragers"). The two data set are thus not fully overlapping, but there is the possibility that some data are present in both sets. In particular, 14 foraging returns collected in 2002 from individuals below 14 years old are present in the `cchunts` data set and could hence have been included in Bird and Bird (*8*). Looking in detail at these subsets, they do not appear to be repetitious (a 9 years old boy present in the `cchunts` dataset does not appear in the Bird and Bird (*8*) study, for example, and none of the younger individuals' returns reported here appear in `cchunts`).

Table S1: **Metadata for included datasets.** These are relative to each source of foraging returns data included in the analysis. The first 11 datasets have been extracted from published papers, the remaining were part of the `cchunts` package. The Data column reports both the code referencing the dataset in our code, e.g. 'Bird_2005' and the reference to the relative study, in italics among parenthesis. As sample size we report the total number of observations for foraging returns, with the total number of foragers under 20 years included in our analysis in parentheses.

Data	Population<br>Years	Resource<br>(Unit)	Ages<br>Sample Size
Bird_2005 (*8*)	Mardu<br>2000-2002	game<br>(kcal/hr)	4- 14<br>157 (22)
BliegeBird_1995 (*35*)	Meriam<br>1993	fish, fruit, mixed<br>(g/min)	3-14<br>12(12)
BliegeBird_2002a (*7*)	Meriam<br>1993-1998	fish<br>(kcal/hr)	4-75<br>196(42)
BlurtonJones_1989 (*17*)	Hadza<br>1985-1986	USO, mixed, fruit<br>(g/hr)	2- 20+<br>70 (29)
BlurtonJones_2002 (*9*)	Hadza<br>1997	USO<br>(kg/hr)	6-75<br>79 (46)

Table S1 continued from previous page

BlurtonJones_1997 (*22*)	Hadza<br>1986- 1989	USO, fruits<br>(kcal/hr)	2-18<br>61 (61)
Bock_2005 (*24*)	Bugakwhe, etc<br>1994	fish<br>(kcal/hr)	5- 14<br>16 (16)
Crittenden_2013 (*10*)	Hadza<br>2005	mixed<br>(kcals/day)	3-17<br>34 (34)
Froehle_2018 (*29*)	Hadza<br>2005	mixed<br>(kcal/trip)	5- 14<br>9 (9)
Hawkes_1995 (*23*)	Hadza<br>1988	USO, fruits<br>(g/hr)	3-17<br>20 (17)
Tucker_2005 (*15*)	Mikea<br>1997- 2003	USO<br>(kcal/hr)	NA<br>254(37)
Alvard_cchunts (*45*)	Piro<br>1989-1991	game<br>(kg/trip)	15-70<br>42(5)
Beckerman_cchunts (*46*)	Bari<br>1970-1972	game<br>(kg/trip)	12-55<br>18(9)
Bird_Bird_Codding_cchunts (*47*)	Mardu<br>2000-2010	game<br>(kg/trip)	7-79<br>77(21)
Coad_cchunts (*48*)	Pouvi, et al<br>2004-2010	game<br>(kg/trip)	15- 69<br>70(7)
Duda_cchunts (*49*)	Baka<br>2012-2013	game<br>(kg/trip)	16- 69<br>57(6)
Ellen_cchunts (*50*)	Nuaulu<br>1970	game<br>(kg/trip)	10-70<br>37(8)
Fernandez_Llamazares_ cchunts (*49*)	Tsimane<br>2012-2013	game<br>(kg/trip)	15-70<br>29(4)
Franzen_cchunts (*51*)	Waorani<br>2002	game<br>(kg/trip)	16- 77<br>48(4)
Gallois_cchunts (*49*)	Baka<br>2012-2013	game<br>(kg/trip)	16- 75<br>80(9)
Gueze_cchunts (*49*)	Punan<br>2012-2013	game<br>(kg/trip)	16- 61<br>35(2)
Headland_cchunts (*52*)	Agta<br>1962-1984	game<br>(kg/trip)	13- 66<br>44(7)
Healey_Nen_PNG_cchunts<br>Preliminary fieldwork	Nen<br>2013	game<br>(kg/trip)	18- 46<br>7(2)
Hill_Kintigh_cchunts (*53*)	Ache<br>1980-2007	game<br>(kg/trip)	11- 75<br>147(37)
Koster_cchunts (*54*)	Mayanga<br>2004-2013	game<br>(kg/trip)	8- 63<br>52(17)
Kramer_Greaves_cchunts (*55*)	Pume'<br>1990-2006	game<br>(kg/trip)	11- 65<br>23(9)
Lupo_Schmitt_cchunts (*56*)	Bofi, Aka<br>1999-2002	game<br>(kg/trip)	6- 60<br>59(20)
Pacheco_cchunts (*57*)	Maya<br>2011-2012	game<br>(kg/trip)	16- 60<br>59(10)
Pangau_Adam_cchunts (*58*)	Nimboran<br>2005-2006	game<br>(kg/trip)	16- 67<br>26(1)
Ready_cchunts (*59*)	Inuit<br>2013-2014	game<br>(kg/trip)	12- 55<br>15(4)
Reyes_Garcia_cchunts (*49*)	Tsimane<br>2012-2013	game<br>(kg/trip)	16- 91<br>37(2)

Table S1 continued from previous page

Sillitoe_cchunts (*60*)	Wola<br>1977	game<br>(kg/trip)	10- 45<br>27(10)
Siren_cchunts (*61*)	Quichua<br>1999, 2008	game<br>(kg/trip)	19- 59<br>2(1)
Trumble_Gurven_cchunts (*11*)	Tsimane<br>2002-2011	game<br>(kg/trip)	7- 82<br>172(53)
Van_Vliet_et_al_Gabon_cchunts (*62*)	Ba-Kota etc<br>2006-2007	game<br>(kg/trip)	15- 45<br>19(3)
Venkataraman_et_al_cchunts (*63*)	Batek<br>1975-1976	game<br>(kg/trip)	9- 50<br>27(11)
Winterhalder_cchunts (*64*)	Cree<br>1975	game<br>(kg/trip)	15- 59<br>16(4)
Yu_et_al_cchunts (*65*)	Matsigenka<br>2004-2007	game<br>(kg/trip)	8- 52<br>69(18)
Ziker_cchunts (*66*)	Dolgan<br>1993- 1997	game<br>(kg/trip)	15- 66<br>26(3)

1.1.1 Excluded datasets

Several papers that passed the first rounds of selection (i.e. appeared to report original data on children foraging returns) were not included in the analysis for a variety of reasons.

Some were subsequently found not to report relevant data (*20, 67*). Others did not include enough data on forager ages (*12, 68–70*). Kramer (*71*) does not include data relative to foraging, and Kramer (*72*) estimates returns from time allocation data, which is not consistent with the other data sets. Several papers reported data in formats that did not allow them to be extracted for analysis, such as failing to report errors around mean return per age class (*5, 73*), or including smoothed loess curves (*74–76*). Kawabe (*42*) reports ranges for individual returns (e.g. "more than 5 specimens killed"). Finally, a number of papers use the same data. In these cases, we included only the data sets for the latest or most informative paper. Bird and Bird (*77*), Blurton Jones (*78*), Pollom et al. (*79*) and Walker (*80*), for example, present data which are best extracted from other papers.

1.1.2 Pooled data

A small subset of the `cchunts` dataset features pooled data (380 out of a total sample size of 2297, representing 17%). For these data points, reported returns are for groups rather than individuals. Social foraging can be conceptualized similarly to technology (guns, dogs) which can increase individual returns. Indeed, including pooled returns has the potential to skew the results by inflating individual returns. To ensure that this is not the case, we first compared the distribution of pooled vs. non pooled data across ages. As can be shown in Figure S14 and S15 respectively, individual and pooled data within the whole data set and by society are evenly distributed across ages, and hence unlikely to bias our results. Moreover, we re-ran the analysis on a subset of data excluding pooled returns. Figure S13 shows that excluding pooled returns does not impact our findings.

1.2 Statistical model

1.2.1 Integrating individual-level data with study-level summary statistics

Our data included a mix of individual-level returns (e.g., a forager brought back y kilograms of fish) and summary statistics, such as the mean and standard deviation of returns for age classes (e.g., children of ages 5-10 collect tubers at a certain average rate). The challenge was to synthesize two distinct types of data: individual-level observations drawn from $f(y|\mu, \sigma)$ and group-level averages $\mathbb{E}[y|\mu, \sigma]$.

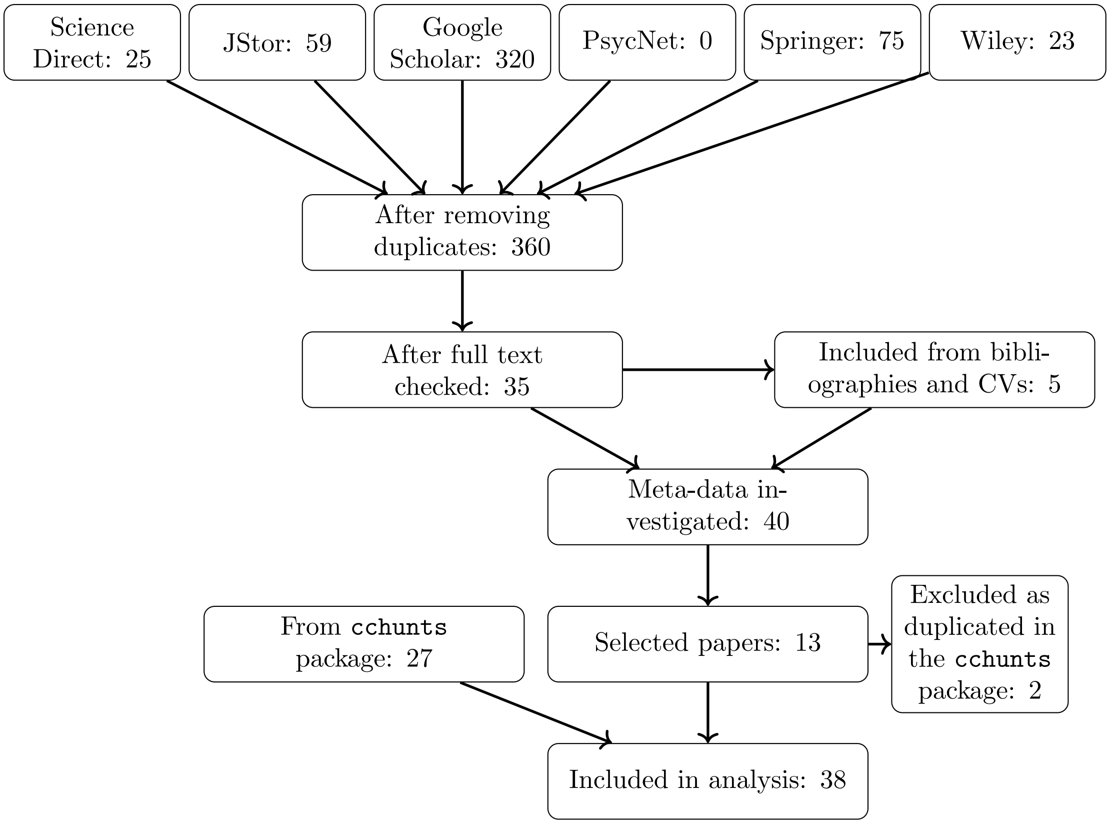

Figure S1: **Process of paper selection.**

When returns were given as summary statistics (i.e., mean and standard error), we modelled them using a measurement error model:

$$\mu_{[\text{obs}]} \sim \text{Normal}(\mathbb{E}[y|\mu,\sigma], \sigma_{\bar{\mu})}$$

Where $\mu_{[\text{obs}]}$ is the group-level mean, $\sigma_{\bar{\mu}}$ is the standard error of that mean, and, following our generative model defined in the main text, $\mathbb{E}[y|\mu,\sigma] = p(\exp(\log(\mu) + \frac{\sigma^2}{2}))$.

Finally, there was some variation in the number of measures available for individuals, and some studies have multiple measurements from the same forager, in which case we included a random effect on skill to account for non-independence of these data points. However, lack of longitudinal data meant that it was not possible to estimate individual differences in the life history parameters.

1.2.2 Priors

We employed regularizing priors for all parameters to reduce over-fitting and facilitate model convergence. Specifically, we assigned a $Normal(0, 1)$ to fixed effects (e.g., intercepts), an $Exponential(1)$ for the random effect standard deviations and $LKJ(2)$ for the correlations between random effects. Moreover, we fixed to zero the starting values for several parameters, as this can be helpful (and in some cases, necessary) for the model start sampling. However, after many iterations of warm up there is no dependence on starting values, unless the model is badly mis-specified or suffers from a multimodal posterior. We used standard MCMC diagnostics and found that all population parameters had Rhat < 1.01 and an effective sample size > 1000.

1.3 Dealing with uncertainty

Uncertainty in age: Forager age was not reported exactly in any study. Most frequently, authors reported an integer age for each child. In other cases an interval of possible ages was given (e.g., 4-7). We modelled age using a Gaussian measurement error model:

$$\text{age}_{\text{obs}} \sim \text{Normal}(\mu_{\text{age}}, \sigma_{\bar{\text{age}}_{\text{obs}}})$$

Uncertainty in sex: In cases where sex of the forager was not reported (or was given as a summary statistic), we average over sex differences in proportion to how often males and females appeared in a given study using Stan's `log_mix()` function.

1.4 Additional figures

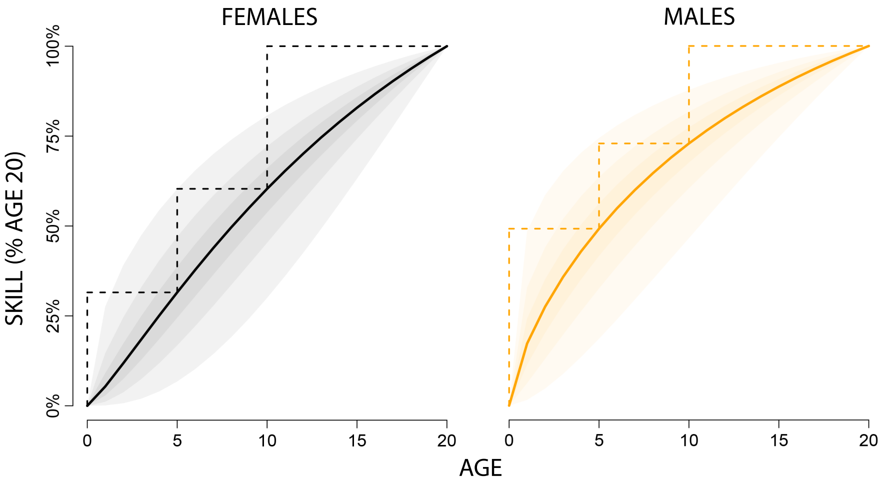

Figure S2: **Predicted change in foraging skill by sex**. Values averaged over variation between studies, individuals, and resource type. The x-axis shows age, while the y-axis is an unit-free measure of the latent variable.

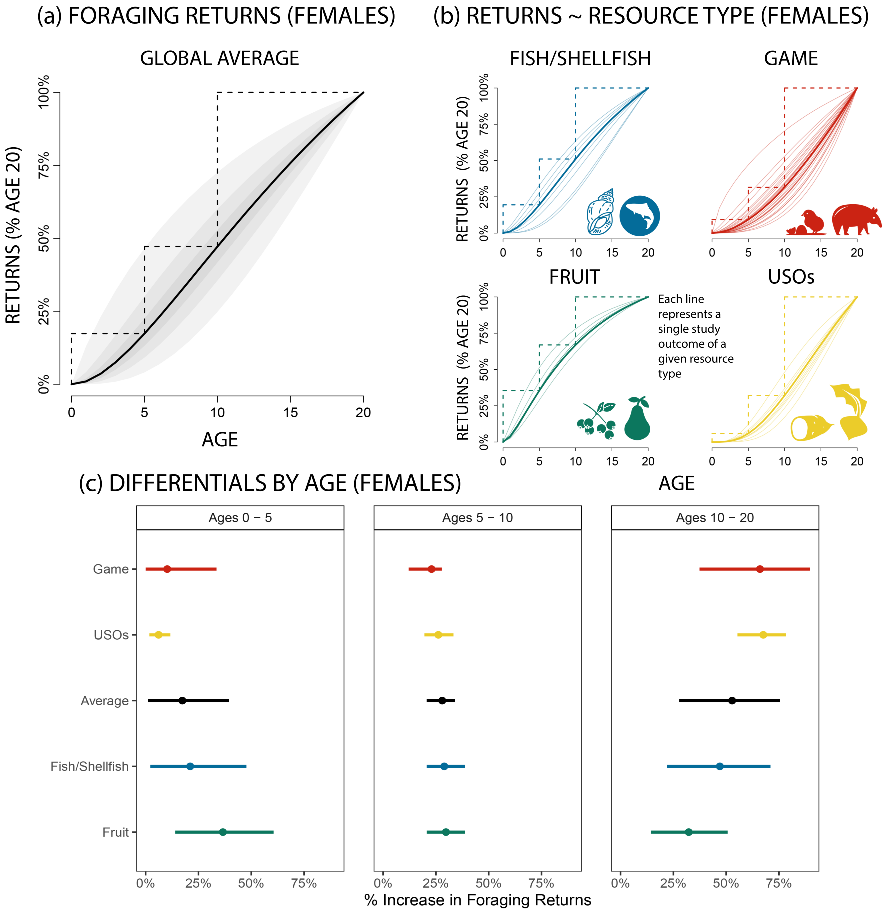

Figure S3: **Foraging returns for females only.** (A)Predicted change in foraging returns with age, averaging over variation between studies, individuals, and resource type. The x-axis shows age, while the y-axis is an unit-free measure of the proportion of increase compared to the maximum value (predicted returns at age 20). Solid line is the posterior median prediction, shaded intervals depict the 30th, 60th, and 90th percentile credible intervals. Dashed lines highlight arbitrary age differentials across childhood. (B) Predicted change in foraging returns by resource type, with thick line denoting the average posterior median and thin lines denoting the median for each unique study outcome for that resource type. All curves are scaled by their maximum value (predicted returns at age 20). The shape of the curves illustrates how productivity increases with age. (C) Percentage increase in foraging returns across childhood, covering the intervals denoted by the dashed lines in A-B. Points indicate posterior median, bars indicate 90% HPDI. This figure is similar to figure 3, but focuses only on females.

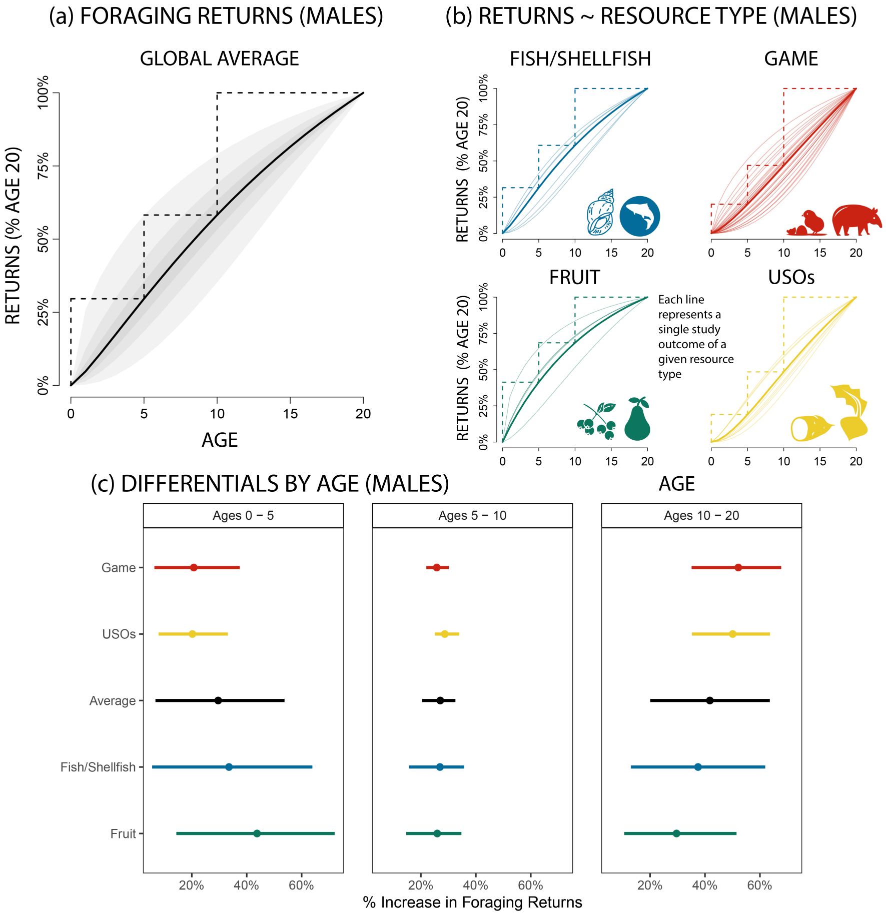

Figure S4: **Foraging returns for males only.** (A)Predicted change in foraging returns with age, averaging over variation between studies, individuals, and resource type. The x-axis shows age, while the y-axis is an unit-free measure of the proportion of increase compared to the maximum value (predicted returns at age 20). Solid line is the posterior median prediction, shaded intervals depict the 30th, 60th, and 90th percentile credible intervals. Dashed lines highlight arbitrary age differentials across childhood. (B) Predicted change in foraging returns by resource type, with thick line denoting the average posterior median and thin lines denoting the median for each unique study outcome for that resource type. All curves are scaled by their maximum value (predicted returns at age 20). The shape of the curves illustrates how productivity increases with age. (C) Percentage increase in foraging returns across childhood, covering the intervals denoted by the dashed lines in A-B. Points indicate posterior median, bars indicate 90% HPDI. This figure is similar to figure 3, but focuses only on males.

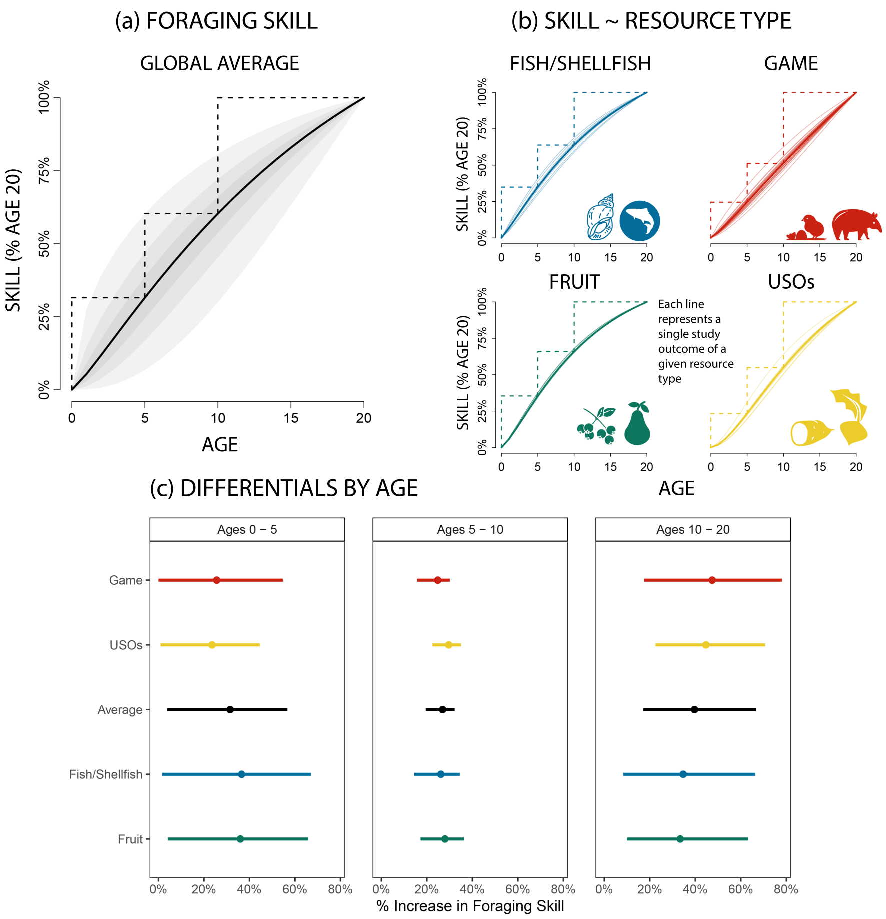

Figure S5: **Foraging skill.** (A)Predicted change in foraging skill with age, averaging over variation between studies, individuals, sex, and resource type. The x-axis shows age, while the y-axis is an unit-free measure of the proportion of increase compared to the maximum value (predicted returns at age 20). Solid line is the posterior median prediction, shaded intervals depict the 30th, 60th, and 90th percentile credible intervals. Dashed lines highlight arbitrary age differentials across childhood. (B) Predicted change in foraging skill by resource type, with thick line denoting the average posterior median and thin lines denoting the median for each unique study outcome for that resource type. All curves are scaled by their maximum value (predicted returns at age 20). The shape of the curves illustrates how skill increases with age. (C) Percentage increase in foraging skill across childhood, covering the intervals denoted by the dashed lines in A-B. Points indicate posterior median, bars indicate 90% HPDI. This figure is similar to figure 3, but describe underlying foraging skill instead of returns.

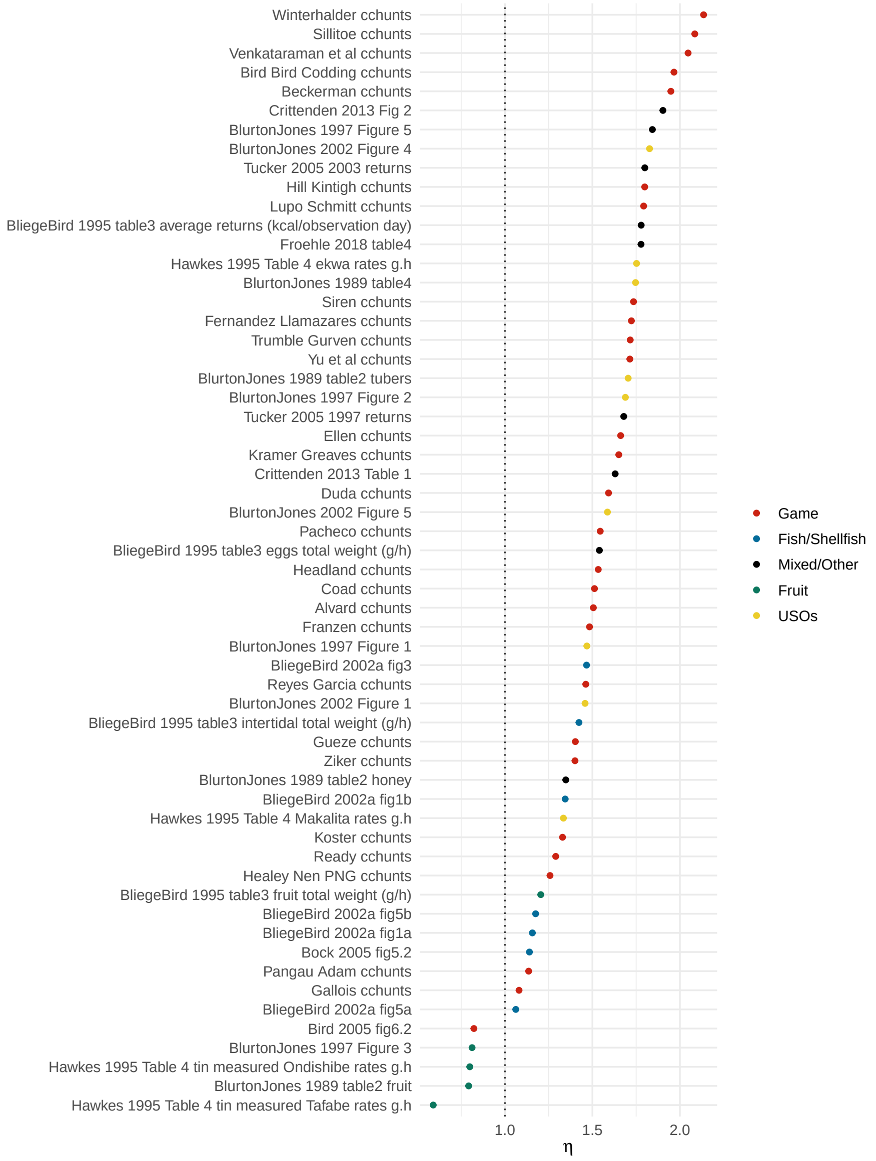

Figure S6: **Mean skill intensity values.** Mean posterior values for η, the skill intensity of foraging, by outcome. Each individual dataset (either present in the `cchunts` package or extracted from a single figure/table) is represented here, color coded for resource.

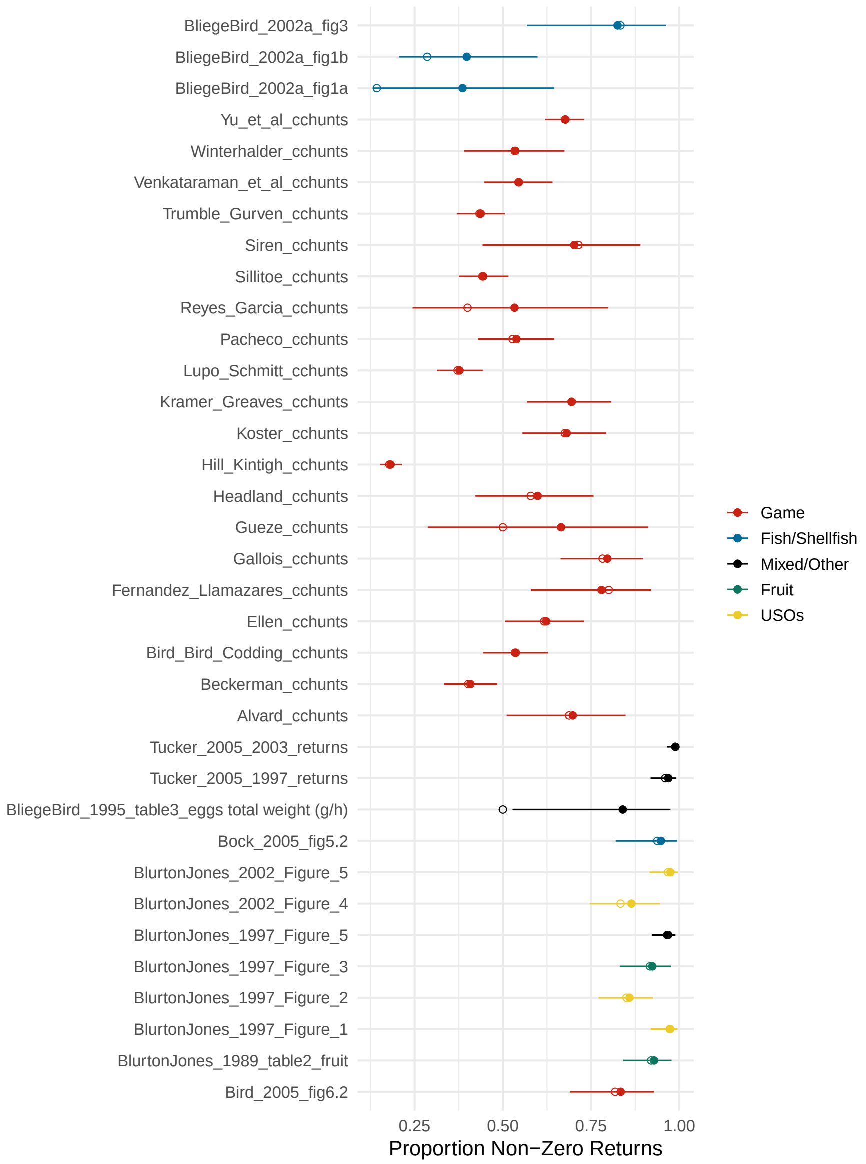

Figure S7: **Real and estimated proportion non-zero returns by outcome.** Empty circles show real proportion of non-zero returns by outcome, mean and 95 percentile intervals of posterior distribution of estimated proportion of non-zero returns are indicated by full circles and bars. The model predicts with sufficient accuracy the proportion of non-zero returns.

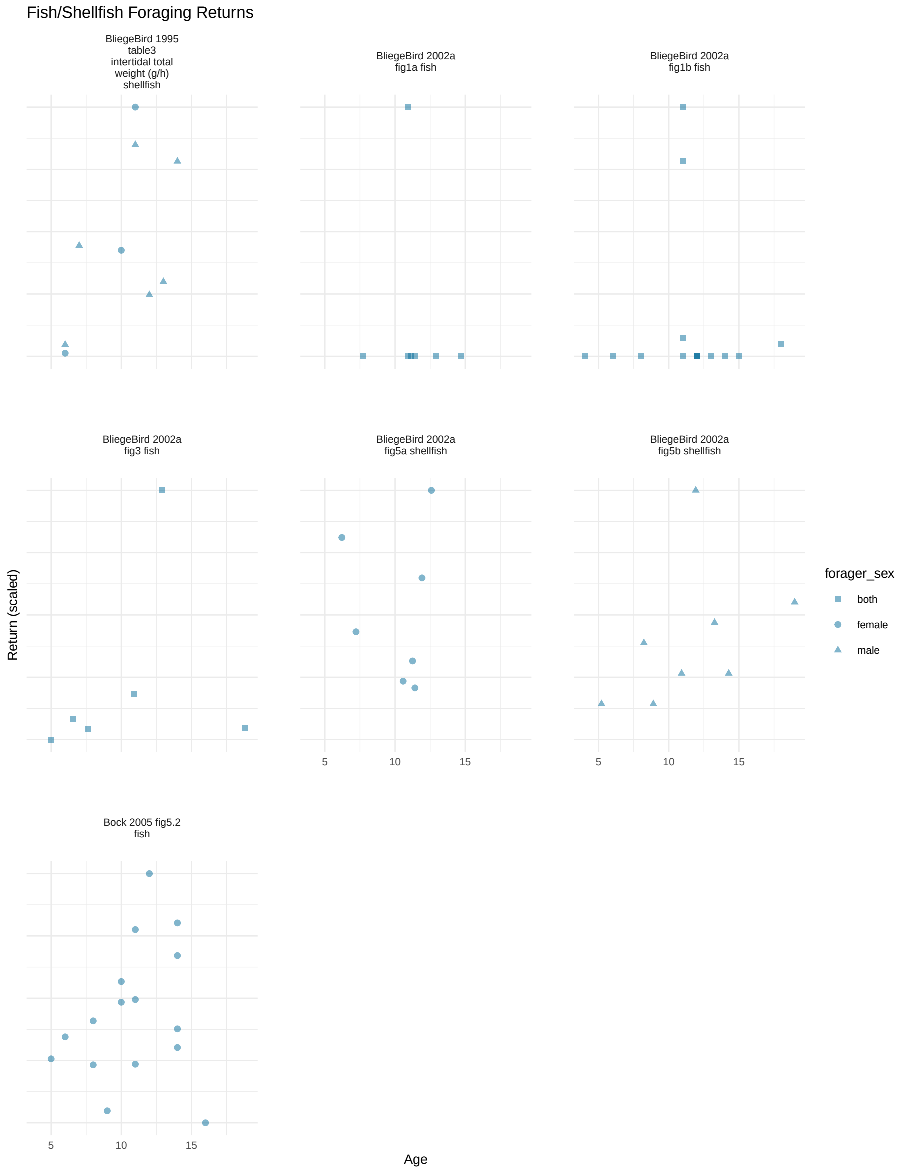

Figure S8: **Fish data.** Data points for all datasets reporting fish and shellfish data.

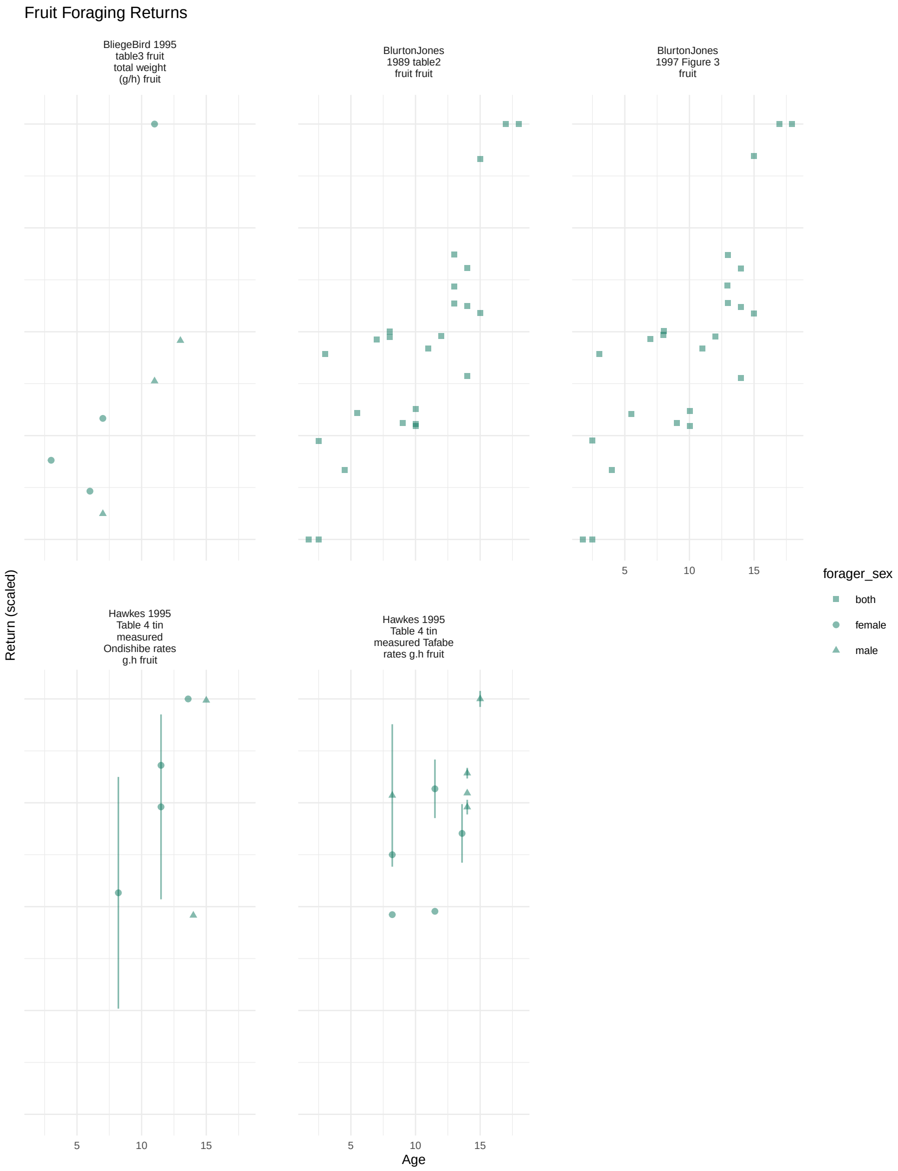

Figure S9: **Fruit data.** Data points for all datasets reporting fruit data.

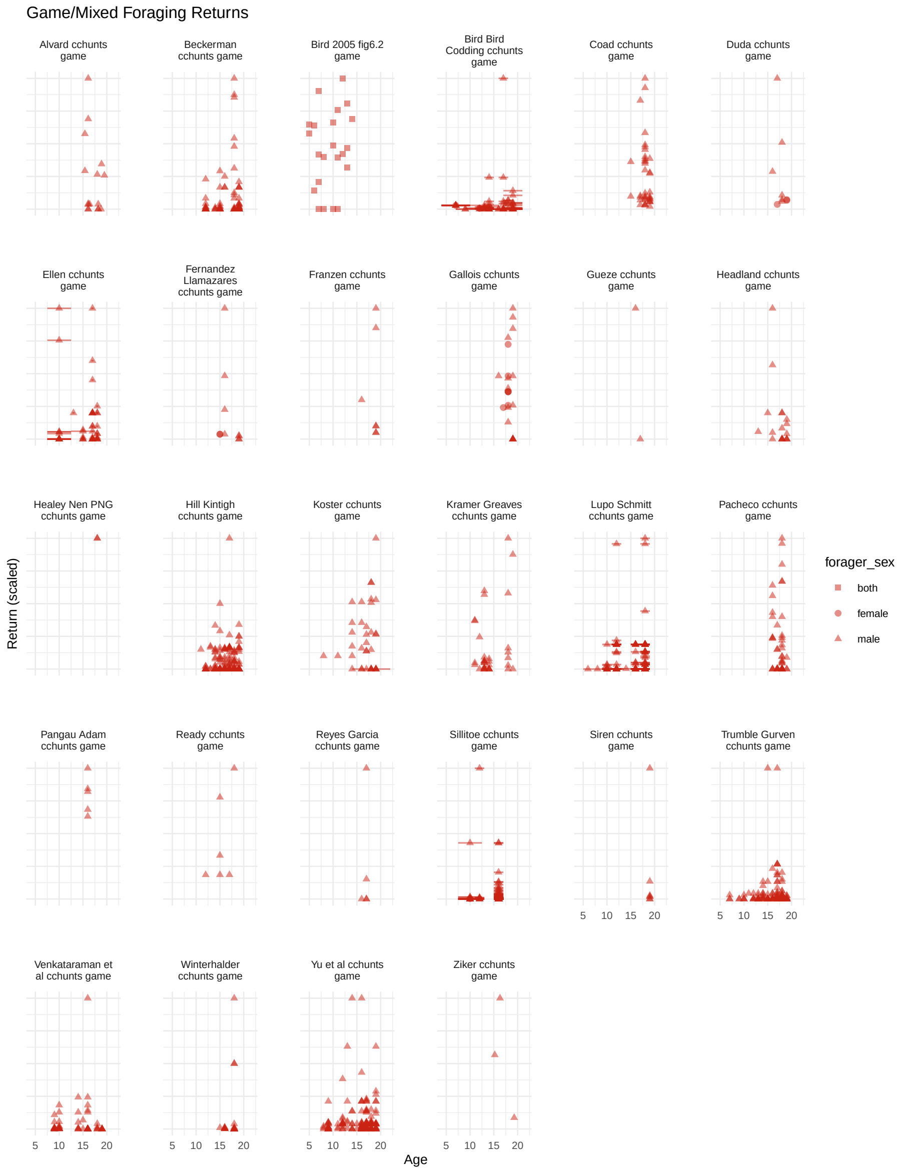

Figure S10: **Game data.** Data points for all datasets reporting game data.

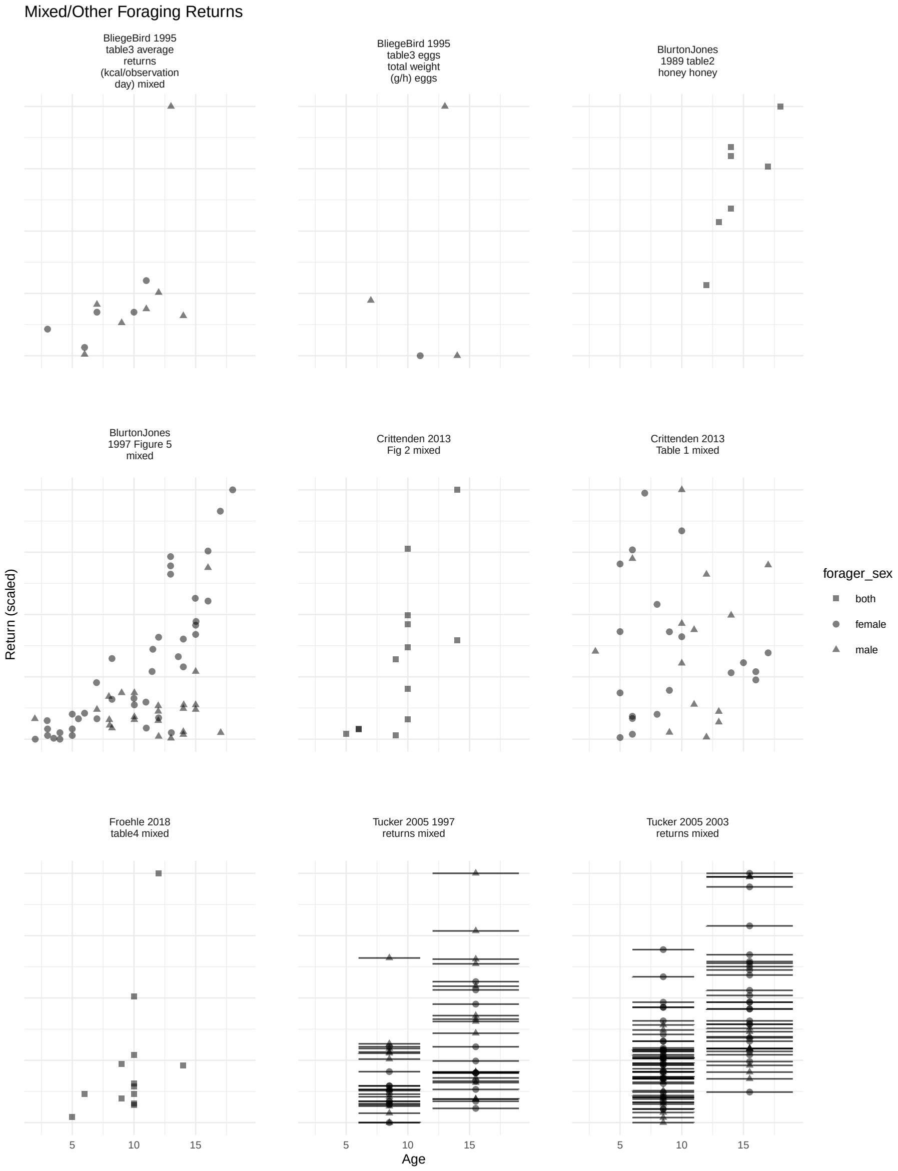

Figure S11: **Data from other resources.** Data points for all datasets reporting data for other kinds of resources.

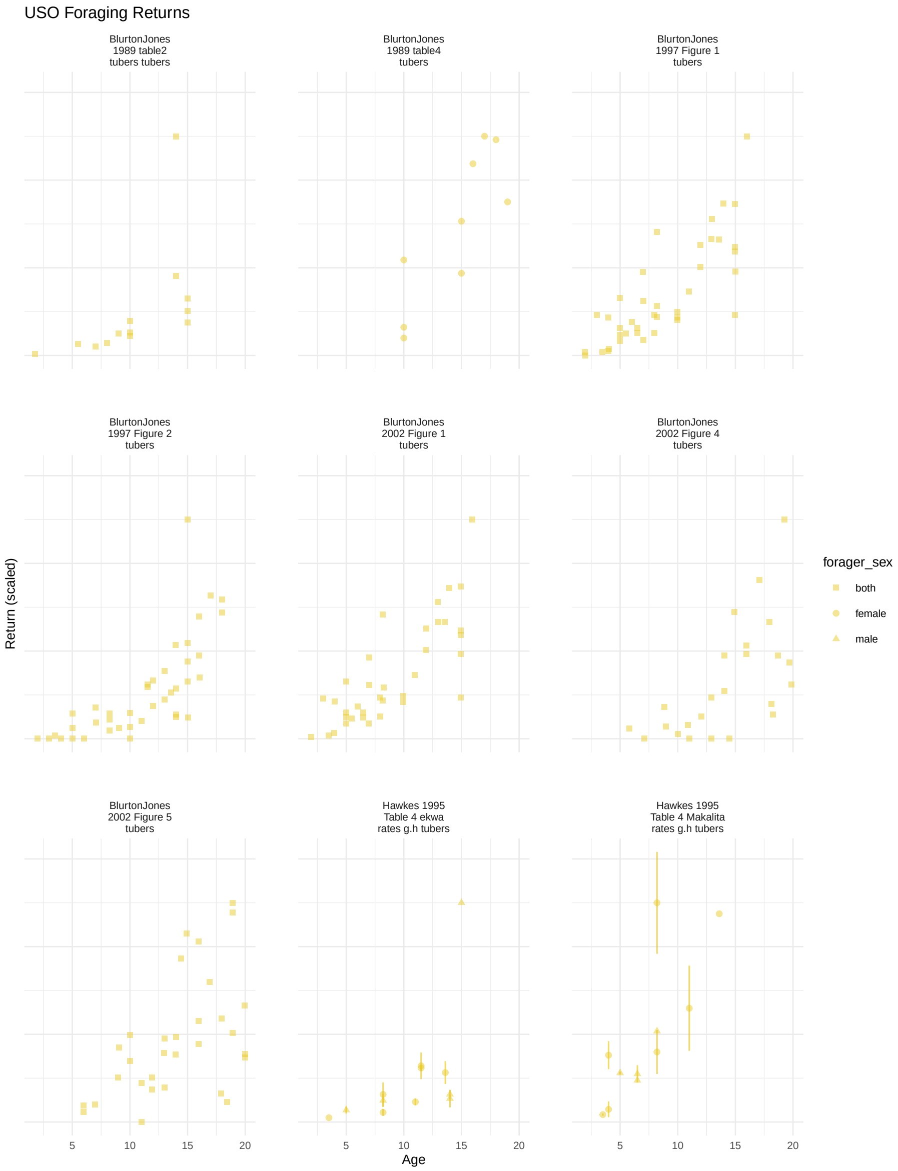

Figure S12: **Tuber data.** Data points for all datasets reporting tuber data.

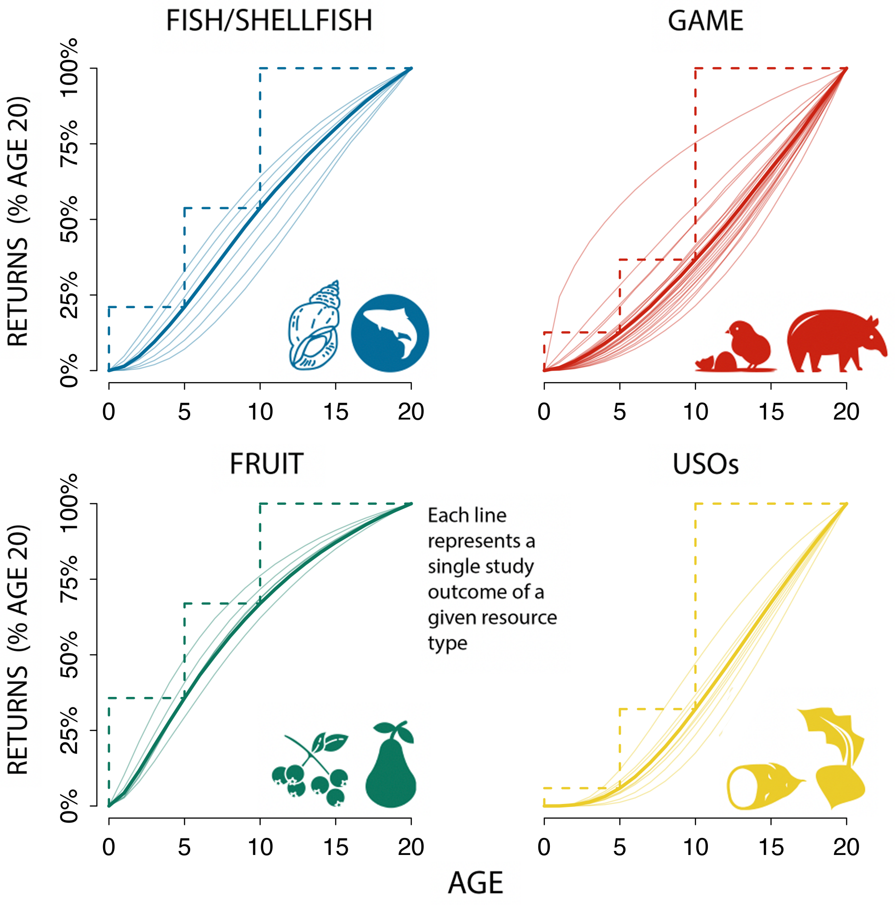

Figure S13: **Returns by age per resource for non-pooled data only.** Predicted change in foraging returns by resource type excluding pooled data in the`cchunts` package, with thick lines denoting the average posterior median and thin lines denoting the median for each unique study outcome for that resource type. All curves are scaled by their maximum value (predicted returns at age 20). Comparing this plot with in-text Figure 3b shows that there is no strong effect of the pooled data on our results.

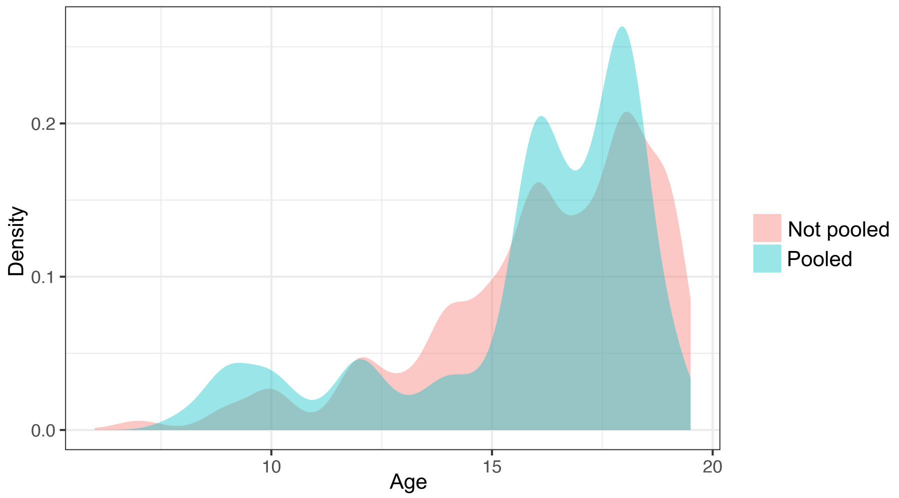

Figure S14: **Data density at each age for pooled vs. non pooled data.**

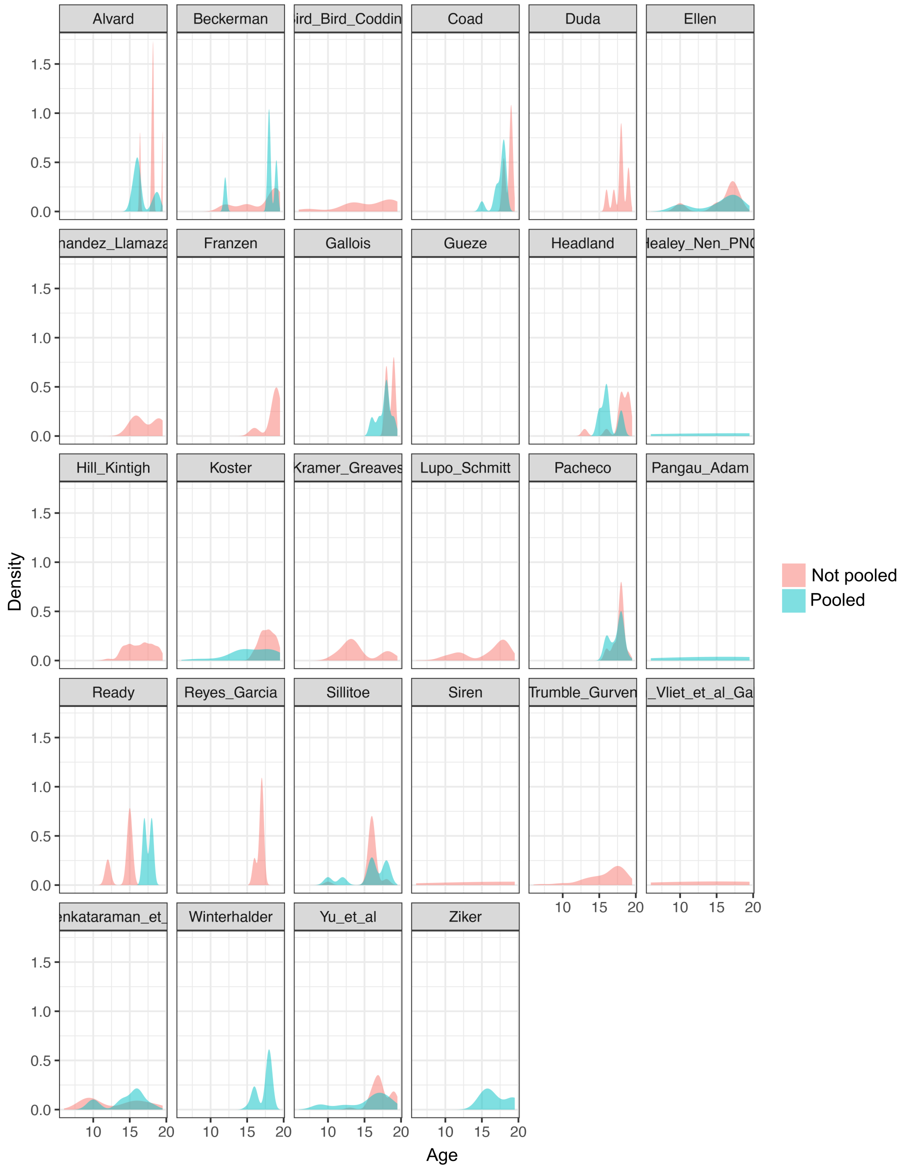

Figure S15: **Data density at each age for pooled vs. non pooled data by population.**

REFERENCES AND NOTES

1. B. Bogin, Evolutionary hypotheses for human childhood. *Am. J. Phys. Anthropol.* **104**, 63–89 (1997).

2. M. Gurven, R. Walker, Energetic demand of multiple dependents and the evolution of slow human growth. *Proc. Biol. Sci.* **273**, 835–841 (2006).

3. E. L. Charnov, D. Berrigan, R. J. H. Bevertron, Dimensionless numbers and the assembly rules for life histories. *Philos. Trans. R. Soc. Lond. B Biol. Sci.* **332**, 41–48 (1991).

4. K. Hawkes, Grandmothers and the evolution of human longevity. *Am. J. Hum. Biol.* **15**, 380 (2003).

5. H. Kaplan, K. Hill, J. Lancaster, A. M. Hurtado, A theory of human life history evolution: Diet, intelligence, and longevity. *Evol. Anthropol.* **9**, 156–185 (2000).

6. K. L. Kramer, P. T. Ellison, Pooled energy budgets: Resituating human energy-allocation trade-offs. *Evol. Anthropol.* **19**, 136–147 (2010).

7. R. B. Bird, D. W. Bird, Constraints of knowing or constraints of growing? *Hum. Nat.* **13**, 239–267 (2002).

8. D. W. Bird, R. B. Bird, Mardu children's hunting strategies in the Western Desert, Australia: Foraging and the evolution of human life histories, in *Hunter Gatherer Childhoods* (AldineTransaction, 2005), pp. 129–146.

9. N. G. Blurton Jones, F. W. Marlowe, Selection for delayed maturity. *Hum. Nat.* **13**, 199–238 (2002).

10. A. N. Crittenden, N. L. Conklin-Brittain, D. A. Zes, M. J. Schoeninger, F. W. Marlowe, Juvenile foraging among the Hadza: Implications for human life history. *Evol. Hum. Behav.* **34**, 299–304 (2013).

11. M. Gurven, H. Kaplan, M. Gutierrez, How long does it take to become a proficient hunter? Implications for the evolution of extended development and long life span. *J. Hum. Evol.* **51**, 454–470 (2006).

12. K. L. Kramer, R. D. Greaves, P. T. Ellison, Early reproductive maturity among Pumé foragers: Implications of a pooled energy model to fast life histories. *Am. J. Hum. Biol.* **21**, 430–437 (2009).

13. J. Koster, R. M. Elreath, K. Hill, D. Yu, G. Shepard Jr., N. van Vliet, M. Gurven, B. Trumble, R. B. Bird, D. Bird, B. Codding, L. Coad, L. Pacheco-Cobos, B. Winterhalder, K. Lupo, D. Schmitt, P. Sillitoe, M. Franzen, M. Alvard, V. Venkataraman, T. Kraft, K. Endicott, S. Beckerman, S. A. Marks, T. Headland, M. Pangau-Adam, A. Siren, K. Kramer, R. Greaves, V. Reyes-García, M. Guèze, R. Duda, Á. Fernández-Llamazares, S. Gallois, L. Napitupulu, R. Ellen, J. Ziker, M. R. Nielsen, E. Ready, C. Healey, C. Ross, The life history of human foraging: Cross-cultural and individual variation. *Sci. Adv.* **6**, eaax9070 (2020).

14. K. L. Kramer, Childhood teaching and learning among Savanna Pumé hunter-gatherers: Mismatch between foraging and postindustrial societies. *Hum. Nat.* **32**, 87–114 (2021).

15. B. Tucker, A. G. Young, Growing up Mikea: Children's time allocation and tuber foraging in southwestern Madagascar, in *Hunter-Gatherer Childhoods* (Aldine Transaction, 2005), pp. 147–171.

16. R. Walker, K. Hill, H. Kaplan, G. McMillan, Age-dependency in hunting ability among the Ache of Eastern Paraguay. *J. Hum. Evol.* **42**, 639–657 (2002).

17. N. G. Blurton Jones, K. Hawkes, J. F. O'Connell, Modelling and measuring the costs of children in two foraging societies, in *Comparative Socioecology, The Behavioural Ecology of Humans and other Mammals* (Blackwell Scientific Publications, 1989), pp. 367–390.

18. A. N. Crittenden, Children's foraging and play among the Hadza, in *Origins and Implications of the Evolution of Childhood* (University of New Mexico Press, 2016), pp. 155–172.

19. N. G. Blurton Jones, K. Hawkes, P. Draper, Differences Between Hazda and !Kung Children's Work: Original Affluence or Practical Reasons, in *Key Issues in Hunther Gatherer Research*, E. S. Burch, L. J. Ellanna, Eds. (Routledge, 1994), pp. 189–215.

20. N. G. Blurton Jones, K. Hawkes, P. Draper, Foraging returns of !Kung adults and children: Why didn't !Kung children forage? *J. Anthropol. Res.* **50**, 217–248 (1994).

21. N. G. Blurton Jones, K. Hawkes, J. F. O'Connell, The global process and local ecology: How should we explain differences between the Hadza and the !Kung? in *Cultural Diversity Among Twentieth-Century Foragers: An African Perspective* (Cambridge Univ. Press, 1996).

22. N. G. Blurton Jones, K. Hawkes, J. F. OConnell, Why do Hazda children forage? in *Uniting Psychology and Biology: Integrative Perspectives on Human Development* (American Psychological Association, 1997), pp. 279–313.

23. K. Hawkes, J. F. O'Connell, N. G. Blurton Jones, Hadza children's foraging: Juvenile dependency, social arrangements, and mobility among hunter-gatherers. *Curr. Anthropol.* **36**, 688–700 (1995).

24. J. Bock, What makes a competent adult forager? in *Hunter-gatherer childhoods* (AldineTransaction, 2005), pp. 109–128.

25. J. B. Lancaster, H. S. Kaplan, K. Hill, A. M. Hurtado, The Evolution of Life History, Intelligence and Diet Among Chimpanzees and Human Foragers, in *Perspectives in Ethology: Evolution, Culture, and Behavior*, F. Tonneau, N. S. Thompson, Eds. (Perspectives in Ethology, Springer US, 2000), pp. 47–72.

26. C. Schuppli, S. M. Graber, K. Isler, C. P. van Schaik, Life history, cognition and the evolution of complex foraging niches. *J. Hum. Evol.* **92**, 91–100 (2016).

27. A. Veile, Hunter-gatherer diets and human behavioral evolution. *Physiol. Behav.* **193**, 190–195 (2018).

28. J. Bock, Learning, life history, and productivity: Children's lives in the Okavango Delta, Botswana. *Hum. Nat.* **13**, 161–197 (2002).

29. A. W. Froehle, G. K. Wells, T. R. Pollom, A. Z. P. Mabulla, S. Lew-Levy, A. N. Crittenden, Physical activity and time budgets of Hadza forager children: Implications for self-provisioning and the ontogeny of the sexual division of labor. *Am. J. Hum. Biol.* **31**, e23209 (2019).

30. S. Lew-Levy, E. J. Ringen, A. N. Crittenden, I. A. Mabulla, T. Broesch, M. A. Kline, The life history of learning subsistence skills among Hadza and BaYaka foragers from Tanzania and the Republic of Congo. *Hum. Nat.* **32**, 16–47 (2021).

31. K. L. Kramer, Variation in juvenile dependence. *Hum. Nat.* **13**, 299–325 (2002).

32. A. K. Basabose, Diet composition of chimpanzees inhabiting the montane forest of Kahuzi, Democratic Republic of Congo. *Am. J. Primatol.* **58**, 1–21 (2002).

33. A. Whiten, R. W. Byrne, R. A. Barton, P. G. Waterman, S. P. Henzi, Dietary and foraging strategies of baboons. *Philos. Trans. R. Soc. Lond. B Biol. Sci.* **334**, 187–197 (1991).

34. T. S. Kraft, V. V. Venkataraman, I. J. Wallace, A. N. Crittenden, N. B. Holowka, J. Stieglitz, J. Harris, D. A. Raichlen, B. Wood, M. Gurven, H. Pontzer, The energetics of uniquely human subsistence strategies. *Science* **374**, eabf0130 (2021).

35. R. B. Bird, D. W. Bird, Children and traditional subsistence on Mer (Murray Island), Torres Strait. *Aust. Aborig. Stud.* **1**, 2 (1995).

36. M. Gurven, H. Kaplan, Determinants of time allocation across the lifespan: A theoretical model and an application to the Machiguenga and Piro of Peru. *Hum. Nat.* **17**, 1–49 (2006).

37. P. Collings, Birth order, age, and hunting success in the Canadian Arctic.*Hum. Nat.* **20**, 354–374 (2009).

38. K. L. Kramer, Cooperative breeding and its significance to the demographic success of humans. *Ann. Rev. Anthropol.* **39**, 417–436 (2010).

39. J. Stieglitz, “Nuclear family conflict and cooperation among Tsimane’ forager -horticulturalists of Bolivia,” thesis, The University of New Mexico, (2009).

40. K. L. Kramer, The evolution of human parental care and recruitment of juvenile help. *Trends Ecol. Evol.* **26**, 533–540 (2011).

41. S. Lew-Levy, R. Reckin, S. M. Kissler, I. Pretelli, A. H. Boyette, A. N. Crittenden, R. V. Hagen, R. Haas, K. L. Kramer, J. Koster, M. J. O'Brien, K. Sonoda, T. A. Surovell, J. Stieglitz, B. Tucker, N. Lavi, K. Ellis-Davies, H. E. Davis, Socioecology shapes child and adolescent time allocation in twelve hunter-gatherer and mixed-subsistence forager societies. *Sci. Rep.* **12**, 8054 (2022).

42. T. Kawabe, Development of hunting and fishing skill among boys of the Gidra in lowland Papua New Guinea. *J. Hum. Ergol.* **12**, 65–74 (1983).

43. J. L. Pick, S. Nakagawa, D. W. A. Noble, Reproducible, flexible and high-throughput data extraction from primary literature: The metaDigitise R package. *Methods Ecol. Evol.* **10**, 426–431 (2019).

44. S. E. Johnson, J. Bock, Trade-offs in skill acquisition and time allocation among juvenile chacma baboons. *Hum. Nat.* **15**, 45–62 (2004).

45. M. Alvard, Shotguns and sustainable hunting in the Neotropics. *Oryx* **29**, 58–66 (1995).

46. S. Beckerman, R. Lizarralde, *The Ecology of the Bar: Rainforest Horticulturalists of South America* (University of Texas Press, 2013).

47. D. W. Bird, R. B. Bird, B. F. Codding, In pursuit of mobile prey: Martu hunting strategies and archaeofaunal interpretation. *Am. Antiq.* **74**, 3–29 (2009).

48. L. M. Coad, "Bushmeat hunting in Gabon: Socio-economics and hunter behaviour," thesis, University of Cambridge (2008).

49. V. Reyes-Garcia, M. Guèze, I. Díaz-Reviriego, R. Duda, Á. Fernández-Llamazares, S. Gallois, L. Napitupulu, M. Orta-Martínez, A. Pyhälä, The adaptive nature of culture: A cross-cultural analysis of the returns of local environmental knowledge in three indigenous societies. *Curr. Anthropol.* **57**, 761–784 (2016).

50. R. Ellen, Individual strategy and cultural regulation in Nuaulu hunting, in *Redefining Nature: Ecology, Culture and Domestication*, R. Ellen, F. Katsuyoshi, Eds. (Routledge, 1996), pp. 597–635.

51. M. Franzen, Evaluating the sustainability of hunting: A comparison of harvest profiles across three Huaorani communities. *Environ. Conserv.* **33**, 36–45 (2006).

52. T. N. Headland, *Why Foragers Do Not Become Farmers: A Historical Study of a Changing Ecosystem and Its Effect on a Negrito Hunter-Gatherer Group in the Philippines* (University of Hawai'i at Manoa, 1986).

53. K. Hill, K. Kintigh, Can anthropologists distinguish good and poor hunters? Implications for hunting hypotheses, sharing conventions, and cultural transmission. *Curr. Anthropol.* **50**, 369–378 (2009).

54. J. M. Koster, Hunting with dogs in Nicaragua: An optimal foraging approach. *Curr. Anthropol.* **49**, 935–944 (2008).

55. K. L. Kramer, R. D. Greaves, Why Pumé Foragers Retain a Hunting and Gathering Way of Life, in *Hunter-gatherers in a Changing World*, V. Reyes-Garca, A. Pyhl, Eds. (Springer International Publishing, 2017), pp. 109–126.

56. K. D. Lupo, D. N. Schmitt, Upper Paleolithic net-hunting, small prey exploitation, and women's work effort: A view from the ethnographic and ethnoarchaeological record of the Congo Basin. *J. Archaeol. Method Theory* **9**, 147–179 (2002).

57. L. Pacheco-Cobos, B. Winterhalder, *Economic Benefits of Hunting with Dogs in the Context of Tropical Horticulture* (2015).

58. M. Pangau-Adam, R. Noske, M. Muehlenberg, Wildmeat or bushmeat? Subsistence hunting and commercial harvesting in Papua (West New Guinea), Indonesia. *Hum. Ecol.* **40**, 611–621 (2012).

59. E. Ready, "Food, sharing, and social structure in an Arctic mixed economy," thesis, Stanford University (2016).

60. P. Sillitoe, *Managing Animals in New Guinea: Preying the Game in the Highlands* (2004).

61. A. H. Sirén, D. S. Wilkie, The effects of ammunition price on subsistence hunting in an Amazonian village. *Oryx* **50**, 47–55 (2016).

62. N. Van Vliet, R. Nasi, Hunting for livelihood in northeast Gabon: Patterns, evolution, and sustainability. *Ecol. Soc.* **13**, 33 (2008).

63. K. Endicott, The hunting methods of the Batek negritos of Malaysia: A problem of alternatives. *Canberra Anthropol.* **2**, 7–22 (1979).

64. B. Winterhalder, Boreal foraging strategies, in *Boreal Forest Adaptations: The Northern Algonkians*, T. A. Steegman, Ed. (Springer, 1983), pp. 201–241.

65. J. Ohl-Schacherer, G. H. Shepard Jr., H. Kaplan, C. A. Peres, T. Levi, D. W. Yu, The sustainability of subsistence hunting by Matsigenka native communities in Manu National Park, Peru. *Conserv. Biol.* **21**, 1174–1185 (2007).

66. J. P. Ziker, *Peoples of the Tundra: Northern Siberians in the Post-Communist Transition* (Waveland Press, 2002).

67. T. S. Kraft, V. V. Venkataraman, I. Tacey, N. J. Dominy, K. M. Endicott, Foraging performance, prosociality, and kin presence do not predict lifetime reproductive success in batek hunter-gatherers. *Hum. Nat.* **30**, 71–97 (2019).

68. D. W. Bird, R. B. Bird, Children on the reef: Slow learning or strategic foraging? *Hum. Nat.* **13**, 269–297 (2002).

69. I. Hagino, T. Yamauchi, High Motivation and Low Gain: Food Procurement from Rainforest Foraging by Baka Hunter-Gatherer Children, in *Social Learning and Innovation in Contemporary Hunter-Gatherers: Evolutionary and Ethnographic Perspectives*, H. Terashima, B. S. Hewlett, Eds. (Replacement of Neanderthals by Modern Humans Series, Springer 2016), pp. 135–144.

70. P. L. Hooper, K. Demps, M. Gurven, D. Gerkey, H. S. Kaplan, Skills, division of labour and economies of scale among Amazonian hunters and South Indian honey collectors. *Phil. Trans. Biol. Sci.* **370**, 20150008 (2015).

71. K. L. Kramer, Production and Consumption across the Life Course, in *Maya Children* (Harvard Univ. Press, 2005), pp. 121–136.

72. K. L. Kramer, Does It Take a Family to Raise a Child? Cooperative Breeding and the Contributions of Maya Siblings, Parents and Older Adults in Raising Children, in *Substitute Parents: Biological and Social Perspectives on Alloparenting in Human Societies*, G. Bentley, R. Mace, Eds. (Berghahn Books, ed. 1, 2009), pp. 77–99.

73. H. S. Kaplan, A. J. Robson, The emergence of humans: The coevolution of intelligence and longevity with intergenerational transfers. *Proc. Natl. Acad. Sci. U.S.A.* **99**, 10221–10226 (2002).

74. H. Kaplan, Evolutionary and wealth flows theories of fertility: Empirical tests and new models. *Popul. Dev. Rev.* **20**, 753 (1994).

75. H. Kaplan, A theory of fertility and parental investment in traditional and modern human societies. *Am. J. Phys. Anthropol.* **101**, 91–135 (1996).

76. F. W. Marlowe, Foraging, in *The Hadza* (University of California Press, ed. 1, 2010), pp. 101–132.

77. D. W. Bird, R. B. Bird, The ethnoarchaeology of juvenile foragers: Shellfishing strategies among Meriam children. *J. Anthropol. Archaeol.* **19**, 461–476 (2000).

78. N. Blurton Jones, The lives of hunter-gatherer children: Effects of parental behavior and parental reproductive strategy, in *Juvenile Primates* (Oxford University Press, 1993), pp. 309–326.

79. T. R. Pollom, K. N. Herlosky, I. A. Mabulla, A. N. Crittenden, Changes in juvenile foraging behavior among the Hadza of Tanzania during early transition to a mixed-subsistence economy. *Hum. Nat.* **31**, 123–140 (2020).

80. R. S. Walker, "Evolution of the human life history: Ontogeny and behavior in two South American indigenous populations," thesis, The University of New Mexico (2004).
